# Evolution and Diversity of a Fungal Self/Nonself Recognition Locus

**DOI:** 10.1371/journal.pone.0014055

**Published:** 2010-11-19

**Authors:** Charles Hall, Juliet Welch, David J. Kowbel, N. Louise Glass

**Affiliations:** Department of Plant and Microbial Biology, University of California, Berkeley, California, United States of America; University of Missouri-Kansas City, United States of America

## Abstract

**Background:**

Self/nonself discrimination is an essential feature for pathogen recognition and graft rejection and is a ubiquitous phenomenon in many organisms. Filamentous fungi, such as *Neurospora crassa*, provide a model for analyses of population genetics/evolution of self/nonself recognition loci due to their haploid nature, small genomes and excellent genetic/genomic resources. In *N. crassa*, nonself discrimination during vegetative growth is determined by 11 *het*erokaryon incompatibility (*het*) loci. Cell fusion between strains that differ in allelic specificity at any of these *het* loci triggers a rapid programmed cell death response.

**Methodology/Principal Findings:**

In this study, we evaluated the evolution, population genetics and selective mechanisms operating at a nonself recognition complex consisting of two closely linked loci, *het-c* (NCU03493) and *pin-c* (NCU03494). The genomic position of *pin-c* next to *het-c* is unique to *Neurospora/Sordaria* species, and originated by gene duplication after divergence from other species within the Sordariaceae. The *het-c pin-c* alleles in *N. crassa* are in severe linkage disequilibrium and consist of three haplotypes, *het-c1*/*pin-c1*, *het-c2/pin-c2* and *het-c3*/*pin-c3*, which are equally frequent in population samples and exhibit trans-species polymorphisms. The absence of recombinant haplotypes is correlated with divergence of the *het-c*/*pin-c* intergenic sequence. Tests for positive and balancing selection at *het-c* and *pin-c* support the conclusion that both of these loci are under non-neutral balancing selection; other regions of both genes appear to be under positive selection. Our data show that the *het-c2/pin-c2* haplotype emerged by a recombination event between the *het-c1*/*pin-c1* and *het-c3*/*pin-c3* approximately 3–12 million years ago.

**Conclusions/Significance:**

These results support models by which loci that confer nonself discrimination form by the association of polymorphic genes with genes containing HET domains. Distinct allele classes can emerge by recombination and positive selection and are subsequently maintained by balancing selection and divergence of intergenic sequence resulting in recombination blocks between haplotypes.

## Introduction

Self/nonself discrimination is a ubiquitous and essential function of both multicellular and microbial species. In vertebrate species, self/non-self recognition relies on the major histocompatibility complex (MHC); allelic polymorphisms at MHC loci in populations are maintained by balancing selection [1], [2]. Alleles at MHC loci show long-term persistence, such that an allele from one species is often more closely related to an allele in a different species, a pattern that is termed *trans-species* polymorphisms. Self/nonself or allorecognition determinants involved in histocompatibility reactions are present in the earliest metazoans, including the ascidian, *Botryllus schlosseri* (Cordate subphylum, Tunicata), and the cnidarium, *Hydractinia symbiolongicarpus* (Phylum Cnidaria) [3]. In plants, self/non-self recognition during sexual reproduction is mediated by the gametophytic or sporophytic self-incompatibility locus, *S*, which elicits recognition and rejection of self-pollen. For example, the *S* locus in crucifers contains two unrelated highly polymorphic recognition genes (*SRK* and *SCR*) that are in tight genetic and physical linkage and encode more than 100 specificities [4]. Alleles at the *S* locus also show trans-species polymorphisms [5].

**Figure 1:**
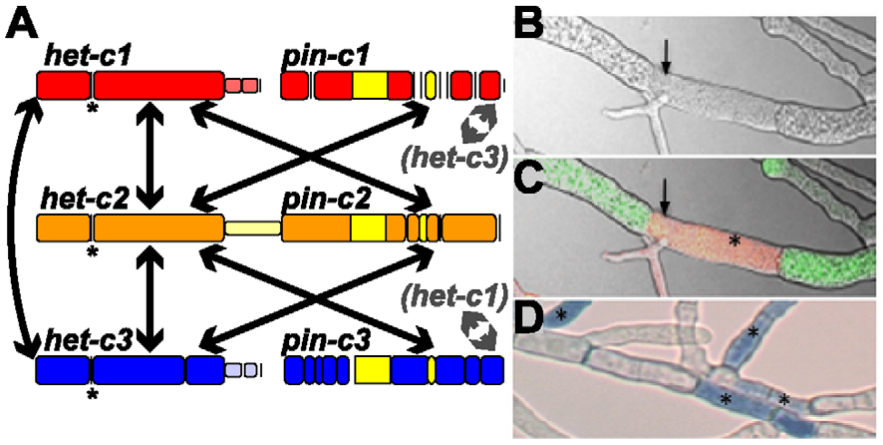
Diagram of the three *het-c/pin-c* haplotypes in *N. crassa* and death of fusion cells as a consequence of heterokaryon incompatibility. A). Cartoon diagram of *het-c1/pin-c1*, *het-c2/pin-c2* and *het-c3/pin-c3* haplotypes. Nonself recognition is mediated by genetic interactions between *het-c* and *pin-c* from alternate haplotypes (*het-c1*-*pin-c2*, *het-c1*-*pin-c3*, *het-c2*-*pin-c1*, *het-c2*-*pin-c3*, *het-c3*-*pin-c1* or *het-c3*-*pin-c2*; arrows). Allelic interactions between alternate *het-c* alleles (*het-c1*-*het-c2*, *het-c1*-*het-c3* or *het-c2*-*het-c3*) is required for a robust HI response [26], [38]; arrows. The allelic specificity domain of *het-c* is indicated by an asterisk ‘*’ [38]. Thinner bars indicate intergenic sequence and indels are represented by open spaces. The HET domain of *pin-c* is shown in yellow. (B–D) Confocal micrographs showing hyphal fusion and heterokaryon formation between a *het-c1/pin-c1* strain bearing cytoplasmic GFP and a *het-c2/pin-c2* strain carrying a nuclear histone HI dsRED marker (isolates are otherwise isogenic). (B) Differential interference contrast (DIC) micrographs. Arrow indicates cell fusion point. (C) merged image of incompatible fusion [7]. Arrow indicates cell fusion point and asterisk indicates compartmentalized hyphal segment. (D) Hyphae stained with the vital dye Evan's blue [89]. Asterisks show compartmentalized dead hyphal segments.

The filamentous fungal ascomycete species, *Neurospora crassa*, is an obligately out breeding haploid organism. Nonself recognition and mating between opposite mating types (*A* and *a*) initiates sexual reproduction. In filamentous fungi, such as *N. crassa*, self/nonself discrimination is also important during vegetative growth. For example, germinating asexual spores and mature hyphae of *N. crassa* colonies undergo cell fusion, which is mediated by self-signaling and chemotropic interactions [6]. However, cell fusion can also occur between colonies of unlike genotypes. If such individuals differ in allelic specificity at nonself recognition loci, the fusion cell is rapidly compartmentalized and undergoes programmed cell death (PCD) (termed heterokaryon incompatibility (HI)) (Figure 1) [7], [8]. HI is suppressed during sexual reproduction in filamentous fungi; genetically different strains are fully inter-fertile and produce viable progeny. HI is analogous to fusion *versus* fusion-rejection in colonial marine invertebrates [9] where encounters between individuals of the same species can lead to fusion to form a single chimeric colony, or rejection, resulting in a histoincompatibility response in colonial invertebrates or HI in filamentous fungi.

HI in filamentous fungi is regulated by genetic loci, termed *het* (for heterokaryon incompatibility) or *vic* (for vegetative incompatibility) [8], [10]. In filamentous fungi, HI has been shown to reduce the risk of transmission of pathogenic elements, such as infectious virus-like dsRNAs [11], [12], [13], exploitation by aggressive genotypes [14] and has been proposed to function in pathogen recognition [15]. The induction of PCD upon nonself recognition in filamentous fungi is extremely rapid and robust; fusion cells are compartmentalized by septa formation and are dead within ∼30 min post-fusion [10], [11]. Ultrastructural and microscopic phenotypes associated with HI share morphological features with PCD in multicellular metazoans [16], [17], [18], including “apoptotic-like” bodies and TUNEL-positive cells, indicative of nuclear degradation. However, mutational analysis of *N. crassa* homologs of genes involved in apoptosis in other eukaryotic species showed that HI and its associated cell death was not dependent upon metacaspase homologs or a homolog to apoptosis inducing factor [19]. These data indicate that HI/PCD in *N. crassa*, and almost certainly in other filamentous fungi, does not occur *via* a conserved apoptotic mechanism, but likely through a fungal-specific pathway.

Genetic dissection of HI systems has been undertaken in a limited number of fungal species, including *N. crassa* (for review, see [7]), *Podospora anserina* (for review, see [8], [15], *Aspergillus nidulans* [20], [21] and *Cryphonectria parasitica* [22]. In *N. crassa*, 11 unlinked loci function in nonself recognition during HI [23]. Two to three allelic specificities occur at each of these loci. Thus, at least 2^11^ different *het* genotypes are possible in *N. crassa* populations. Of these eleven *het* loci, the *het-c* locus, comprised of two closely linked loci, *het-c* and *pin-c*, is the best characterized on a molecular and genetic level. The *het-c* gene encodes a glycine-rich single-pass plasma membrane protein [24], [25]. The *pin-c* gene encodes a protein with a HET domain (pfam PF06985 [26]). Three *het-c pin-c* haplotypes have been identified; *het-c1*/*pin-c1* (formerly known as *het-c^OR^*, Oak Ridge), *het-c2/pin-c2* (formerly known as *het-c^PA^*, Panama) and *het-c3*/*pin-c3* (formerly known as *het-c^GR^*, Groveland). Nonself discrimination between strains that differ in *het-c*/*pin-c* haplotype requires both allelic (*het-c/het-c*) and non-allelic interactions (between *het-c* and *pin-c*) (Figure 1) [26].

In this study, we examined the evolution of the *het-c/pin-c* region within ascomycete fungi and asked whether balancing selection extends across the entire *het-c/pin-c* region. Novel regions of *het-c* and *pin-c*, which appear to be under balancing or positive selection, were identified. We subsequently examined hypotheses as to how linkage disequilibrium of the different *het-c/pin-c* haplotypes may be maintained in populations. Our findings indicate that composition of *het* loci and the number of alleles found at each *het* locus within a species is variable and lineage specific, thus shedding new light on the mechanisms by which self/nonself discrimination loci evolve.

## Results

### Analysis of gene order and the origin of *pin-c*

**Figure 2:**
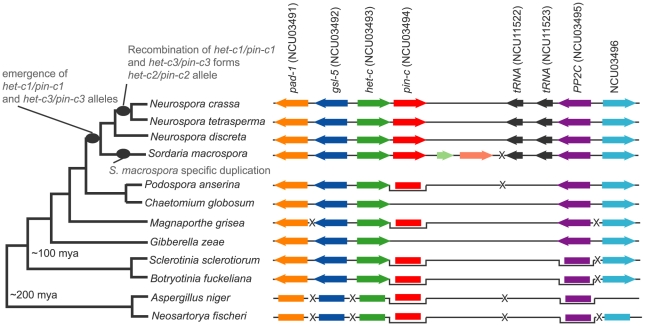
Analysis of gene order shows the recent acquisition of *pin-c* in the *het-c* region in *Neurospora/Sordaria*. Bars indicate genes and are not drawn to scale. Arrows indicate gene orientation. Lines connect genes that are immediate neighbors on a chromosome or contig. Colored boxes indicate homologous genes as determined by sequence conservation and conserved gene order. Genes not connected to lines are found on a different chromosome or contig than the rest and are therefore unlinked. ‘X’ indicates genome rearrangement break points or indicates presence of intervening gene(s) (not shown). The *het-c/pin-c* (NCU03493/NCU03494) region in *S. macrospora* has undergone a gene duplication and rearrangement [29], resulting in one full and one partial *het-c* paralogs and two complete and divergent *pin-c* paralogs. The cladogram to the left represents the evolutionary relationship between species and is derived from James *et al.* [90]. Estimated divergence times are derived from Taylor and Berbee [34].

**Figure 3:**
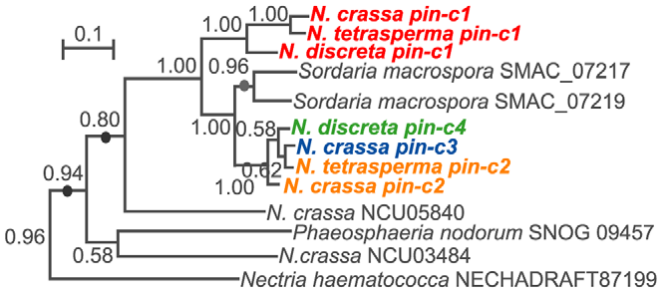
Bayesian inference of the amino acid sequence of *pin-c* alleles and related proteins shows three independent duplication events. Numbers indicate support for nodes based on posterior probability (Bayesian inference). Sequences of *pin-c* (NCU03494) proteins are in bold and in color, reflecting allelic specificity. Circles on branches indicate possible gene duplication events. An expanded phylogeny is provided as Figure S1.

Previously, it was shown that the *het-c* allelic specificity region (∼200 bp) exhibits trans-species polymorphism [27]. We hypothesized that *het-c/pin-c* may have evolved as a *het* locus in *Neurospora* and closely related genera in the Sordariaceae as a result of a genome rearrangement. To test this hypothesis, we aligned a ∼40 k.bp. region that encompassed *het-c* from the genome sequences of *N. crassa* [28], *Neurospora tetrasperma*, *Neurospora discreta*, *Sordaria macrospora* [29], *P. anserina* [30], *Chaetomium globosum*, *Magnaporthe grisea* [31], *Gibberella zeae*, *Sclerotinia sclerotiorum*, *Botryotinia fuckeliana*, *Histoplasma capsulatum*, *Aspergillus niger* [32] and *Neosartorya fischerii* [33] (See Materials and Methods) (Figure 2). With the exception of *pin-c* (NCU03494), gene order and content in this region was well conserved among filamentous ascomycete species. Synteny analysis supported an ancestral gene order of RNA splicing factor *Pad-1* (NCU03491), fatty acid hydroxylase *gsl-5* (NCU03492) and protein phosphatase 2C (NCU03495). The placement of *het-c* (NCU03493) next to *gsl-5* (NCU03492) is ancient and occurred during the divergence of the Sordariomycota from the Eurotiomycota (approximately 250–600 MYA [34]). The *het-c* (NCU03493) and *pin-c* (NCU03494) genes are linked only in closely related species within the Sordariaceae (*Neurospora* and *Sordaria*). In the *Neurospora* lineage, genes of the *pin-c* family have undergone at least two duplications from the ancestral gene (Figure 3; See Figure S1 for expanded phylogeny) resulting in NCU05840, NCU03484 and *pin-c* (NCU03494). These results were surprising because *N. crassa* does not tolerate gene duplications due to a genome defense mechanism termed Repeat-induced Point (RIP) mutation [35]. Evidence for RIP in NCU05840, NCU03484 and *pin-c* loci was not detected (data not shown). After the divergence of the Sordariaceae from the rest of the Sordariomycota, the NCU05840 gene duplication and rearrangement led to the current placement of *pin-c* next to *het-c* (Figure 2). A comparison of the *het-c/pin-c* region between *N. crassa* and the homothallic species, *S. macrospora*, showed evidence for two tandem duplications [29] resulting in two divergent *pin-c* alleles and one *het-c* allele. It is possible that the rearrangements occurred at the tRNA genes found between *pin-c* (*S. macrospora* SMAC07229) and PP2C (*S. macrospora* SMAC07230). In other species, tRNA genes are major sites of rearrangement in repeat poor genomes [28], [36]. In non-Sordariaceae members of the Sordariomycota, the homolog(s) of *pin-c* are in a different genomic location(s) and are not orthologous to *pin-c* or to NCU03484. Unlike the phylogeny of *het-c* homologs in the Pezizomycotina [37], the phylogeny of NCU05840/NCU03494/NCU03484 homologs in filamentous ascomycete fungi was not congruent with species relationships (Figure S1). These observations indicate that multiple rounds of gene duplication and gene loss occurs in this family, as previously observed for HET domain containing genes in species within the Aspergilli [37].

### *het-c* (NCU03493) and *pin-c* (NCU03494) show trans-species polymorphism and are in severe linkage disequilibrium

**Figure 4:**
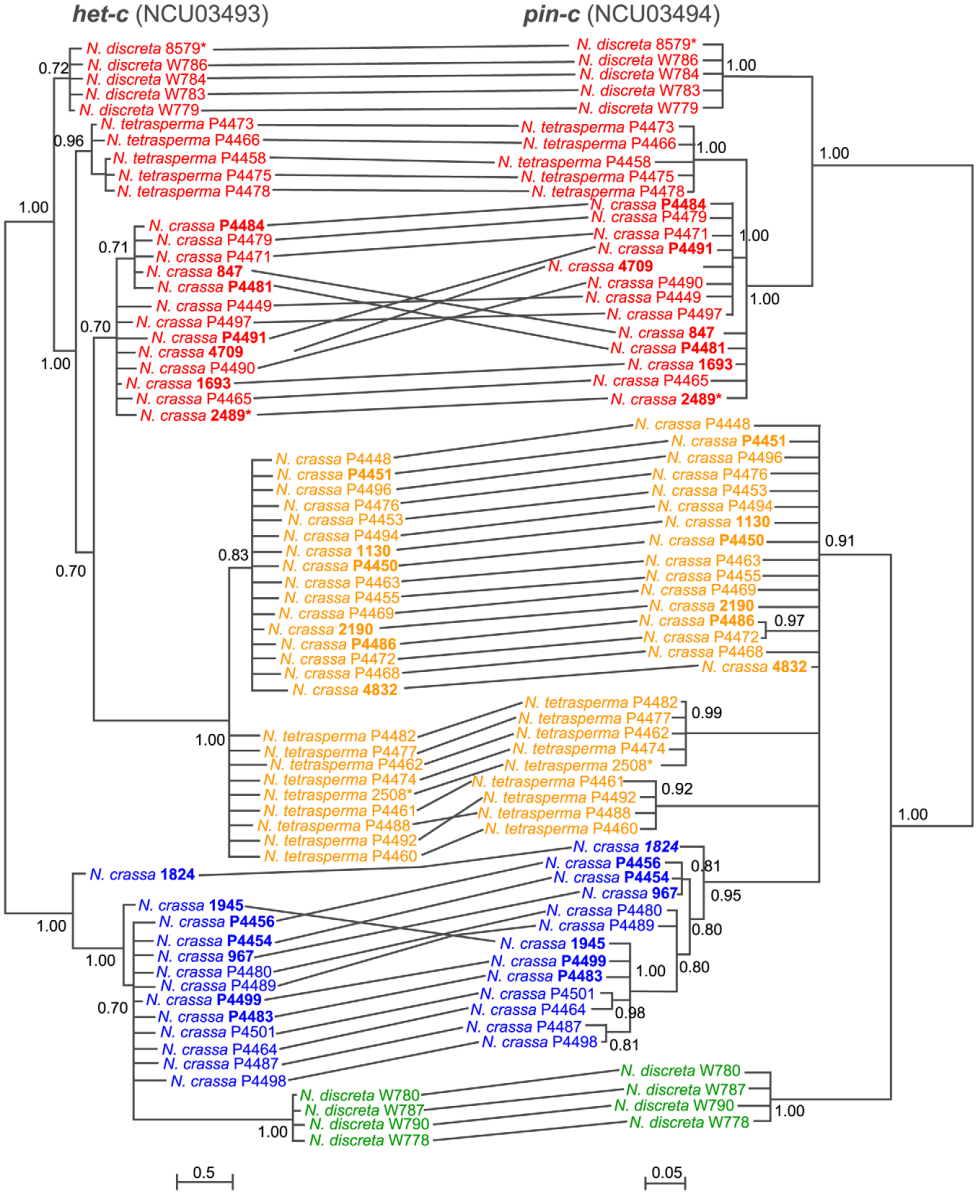
*het-c* (NCU03493) and *pin-c* (NCU03494) show trans-species polymorphism and are in severe linkage disequilibrium. Trees are derived from alignments of partial sequences that correspond to allele specificity domains and were derived by Bayesian analysis [91]. Bayesian posterior probabilities are presented at internal branches. For each clade, alleles are in red, yellow, and blue are for *het-c1/pin-c1*, *het-c2/pin-c2* and *het-c3/pin-c3*, respectively. *Neurospora discreta* has two haplotypes, one clearly related to *het-c1*/*pin-c1* (red), but the other is ambiguous relative to *het-c2/pin-c2* and *het-c3*/*pin-c3*. This haplotype is marked by a green color. In all three *Neurospora* species, *het-c* and *pin-c* alleles show severe linkage disequilibrium (i.e. *het-c1* is always with *pin-c1*, *het-c2* is always with *pin-c2* and *het-c3* is always with *pin-c3*). Lines connecting alleles between the two trees highlight this correspondence. Crossed lines indicate possible intra-allelic recombination events. Taxa in bold indicate that *het-c pin-c* allelic specificity was confirmed by functional tests (Table S1).

Functional analysis of *het-c* showed that allelic specificity is conferred by a ∼34–48 amino acid region characterized by allele-specific indel patterns that show balancing selection [27], [38], [39]. To extend this study on *het-c* and to determine whether *pin-c* is also under balancing selection, we evaluated the *het-c* specificity domain and a highly variable region of *pin-c* from 42 strains of *N. crassa*, 9 strains of *N. discreta*, and 14 strains of *N. tetrasperma* (Table S1). All 42 strains of *N. crassa* contained only one of the three previously characterized *het-c* alleles, which were present at nearly equal frequency in both global (all strains used in this study) and on a local population scale (Louisiana isolates, all from Franklin, LA) (Figure 4; Figure S2). Analysis of the variable region of *pin-c* showed an identical pattern (Figure 4; Figure S2) with three classes of *pin-c* alleles present at nearly equal frequency in both global and local population samples. In contrast to *N. crassa*, only two allele classes of both *het-c* and *pin-c* were recovered from *N. discreta* and *N. tetrasperma* isolates (Figure 4). Most strikingly, in all three species, *het-c* and *pin-c* alleles were in severe linkage disequilibrium: a particular allele of *het-c* was associated with the corresponding allele at *pin-c*. In *N. crassa*, this corresponds to the *het-c1/pin-c1*, *het-c2/pin-c2* and *het-c3/pin-c3* haplotypes (red, yellow and blue, respectively, Figure 4 [26]). In *N. tetrasperma*, only *het-c1/pin-c1* and *het-c2/pin-c2* haplotypes were identified, consistent with previous data for *het-c* [40]. *N. discreta* also possessed only two haplotypes: a *het-c1*/*pin-c1* haplotype, as well as a haplotype (*het-c4/pin-c4*) that was distinct (Figure 4; green).

**Figure 5:**
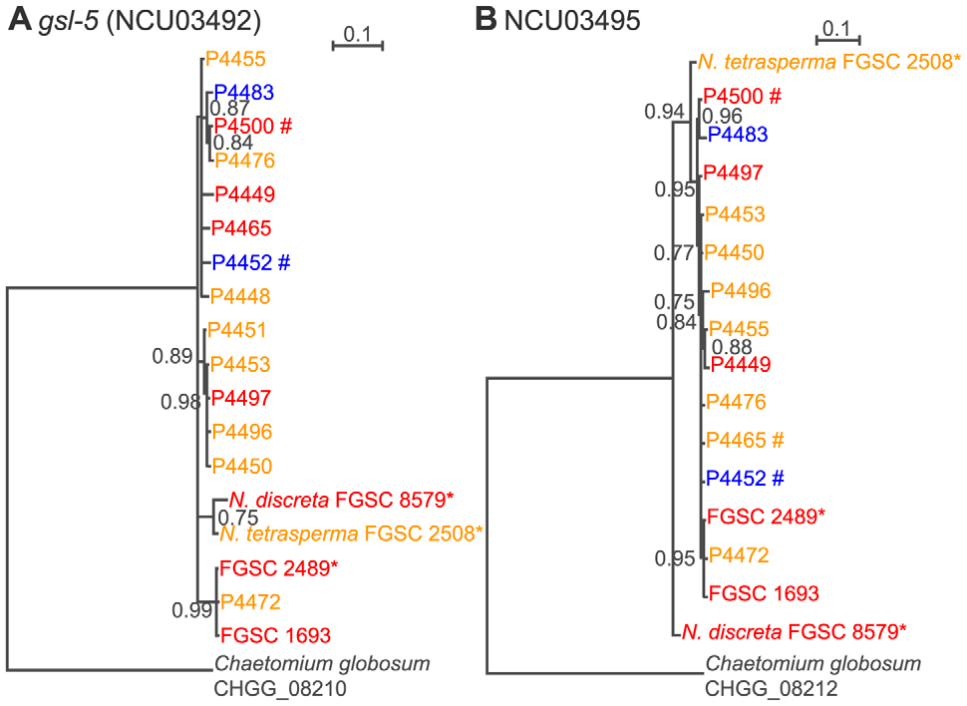
Gene phylogenies of *gsl-5* (NCU03492) or PP2C (NCU03495) show no evidence of linkage disequilibrium with *het-c* (NCU03493) or *pin-c* (NCU03494). Trees were derived from mRNA sequences assembled from RNA-Seq data from isolates shown by Bayesian analysis [91]. Bayesian posterior probabilities are presented at internal branches. Colors represent specificity based on *het-c/pin-c* haplotype (red: *het-c1/pin-c1*, yellow: *het-c2/pin-c2*, blue: *het-c3/pin-c3*). Alleles without species names are *N. crassa*. Alleles from sequenced strains are marked with ‘*’. Alleles not found in Figure 4 are marked with ‘#’. Note that allelic differences at NCU03492 and NCU03495 do not group with allelic specificity at NCU03493 *(het-c)* or NCU03494 *(pin-c)*, indicating that balancing selection and linkage disequilibrium do not extend beyond *het-c* and *pin-c*.

The maintenance of balancing selection in most species is associated with a strong recombination block and extreme sequence divergence [41], [42]. In filamentous fungi, this phenomenon is best demonstrated in the *het-6* locus of *N. crassa*. Like *het-c*, *het-6* HI is controlled by two closely linked genes: *het-6* and *un-24* [43], with two distinct allele specificities [44]. Alleles at *un-24* and *het-6* show severe linkage disequilibrium due to a chromosomal inversion that blocks recombination in this region [43], [45]. If *het-c* and *pin-c* were in a region that was associated with a chromosomal rearrangement, we hypothesized that alleles at loci linked to *het-c/pin-c* would show linkage disequilibrium, or possibly evidence of a chromosomal rearrangement(s). Increased genetic diversity at closely linked neutral sites is often observed near loci under balancing selection [46]. We first examined the linkage of loci surrounding *het-c* and *pin-c* in all three haplotypes *via* PCR amplification and DNA sequencing. All three haplotypes showed an identical gene order surrounding *het-c/pin-c* (data not shown), indicating that a chromosomal rearrangement is not present at the centromere proximal or distal ends of *het-c/pin-c*. To determine whether alleles at the centromere-proximal locus, *gsl-5* (NCU03492; 1.8 kbp from *het-c*) or at the centromere distal locus, NCU03495 (2 kbp from *pin-c*), were in linkage disequilibrium with *het-c/pin-c* haplotypes, we used RNA-Seq data from *N. crassa* strains used in this study to construct gene phylogenies of *gsl-5* (NCU03492) and NCU03495. As shown in Figure 5, a comparison of the topology of *gsl-5* (NCU03492) and NCU03495 showed that alleles from neither locus is in linkage disequilibrium with alleles at *het-c* (NCU03493) or *pin-c* (NCU03494), nor do they show evidence of trans-species polymorphisms. These data indicate that the recombination block observed in the *het-c/pin-c* haplotypes is restricted to these two loci and does not extend to surrounding genes.

To determine whether the divergence in the intergenic region of *het-c/pin-c* may contribute to a recombination block, we sequenced the intergenic region from 35 strains (22 *N. crassa*, 4 *N. discreta*, and 9 *N. tetrasperma*). Each *het-c/pin-c* haplotype contained a unique intergenic sequence profile (Figure S3). The intergenic sequences not only varied in length (Table 1), but also in the level of nucleotide substitution (Table 2). For example, a 177 bp insertion of the coding sequence of another gene, NCU08027 (encoding a nucleoside diphosphatase) was detected in the *N. crassa het-c2/pin-c2* haplotype (Figure S3). Pair-wise measurements of nucleotide diversity between haplotypes (π [47]) showed a 10-fold difference in nucleotide diversity between *versus* within haplotypes (Table 2). These data support the hypothesis that recombination between *het-c* and *pin-c* is suppressed by DNA sequence divergence, particularly in the *het-c/pin-c* intergenic region.

**Table 1:** Length of *het-c* (NCU03493)*/pin-c* (NCU03494) intergenic sequences.

Haplotype	Intergenic sequence length (base pairs)
*N. crassa het-c1/pin-c1*	978
*N. crassa het-c2/pin-c2*	1476
*N. crassa het-c2/pin-c2* variant (FGSC 4832)	1047
*N. crassa het-c3/pin-c3*	919
*N. discreta het-c1/pin-c1*	1811
*N. discreta het-c4/pin-c4*	863
*N. tetrasperma het-c1/pin-c1*	973
*N. tetrasperma het-c2/pin-c2*	920

**Table 2:** The average number of nucleotide differences per site (π) for pair wise comparisons of *N. crassa het-c* (NCU03493)*/pin-c* (NCU03494) intergenic haplotypes.

	*het-c1/pin-c1*	*het-c2/pin-c2*	*het-c2/pin-c2* variant (FGSC 4832)	*het-c3/pin-c3*
***het-c1/pin-c1***	0.04929	0.09498	0.10672	0.16134
***het-c2/pin-c2***	-	0.05876	0.05168	0.20248
***het-c2/pin-c2*** ** variant (FGSC 4832)**	-	-	0	0.19775
***het-c3/pin-c3***	-	-	-	0.02966

### Tests of selection of *het-c* and *pin-c* show that both genes are under both balancing and positive selection

Methods used to detect selection on coding sequences fall into two major classes: population methods, based on analyzing the nature and frequency of allele diversity within a species, and codon analysis methods, based on comparing patterns of synonymous and non-synonymous changes in protein coding sequences. High non-synonymous/synonymous substitution ratios have been observed in *N. crassa het-c* [27] and *het-6* [43], as well as *het* loci from *P. anserina* [48], [49]. Among population-based methods, Tajima's D is an indicator of coding sequences (CDS) evolving under a non-random process, including directional selection or balancing selection [50]. In a stable population at equilibrium, Tajima's D for a CDS should be close to zero. A positive Tajima's D indicates a contraction in population size or balancing selection, which result in low levels of both rare and high frequency polymorphism, while a negative Tajima's D is associated with positive or diversifying selection, population size expansion or recovery from a selective sweep.

We calculated Tajima's D statistic for *het-c* (NCU03493) and *pin-c* (NCU03494) within allele classes and between allele classes for 29 *N. crassa* strains from Franklin, Louisiana (Table 3). As expected, a positive D statistic was obtained when all three allele classes of *het-c* were analyzed, consistent for a locus under balancing selection. In contrast, analysis of each individual *het-c* allele class resulted in a significant negative D score for *het-c2* and *het-c3* (P-value<0.05, all P-values for Tajima's D scores calculated using assumed beta distribution [50]) and a borderline significant (P-value<0.1) score for *het-c1*, suggesting that each *het-c* allele class is also under directional selection. For all *pin-c* alleles, a significantly positive D score of 2.45 (P-value<0.05) was obtained, providing strong evidence for balancing selection acting at this locus. For individual allele classes of *pin-c*, although variation in the D statistic was observed, it was not statistically significant (all P-values>0.1).

**Table 3:** Tajima's D statistic for coding regions within and between *het-c* (NCU03493) and *pin-c* (NCU03494) allele classes.

Allele	Tajima's D statistic	Probability
***het-c*** **, all alleles**	1.33965	P>0.10
*het-c1* alleles	−1.53263	0.10>P>0.05
*het-c2* alleles	−1.90353	P<0.05*
*het-c3* alleles	−2.23236	P<0.01*
***pin-c*** **, all alleles**	2.44820	P<0.05*
*pin-c1* alleles	1.15021	P>0.10
*pin-c2* alleles	−1.04141	P>0.10
*pin-c3* alleles	0.09330	P>0.10

* = significant value.

**Figure 6:**
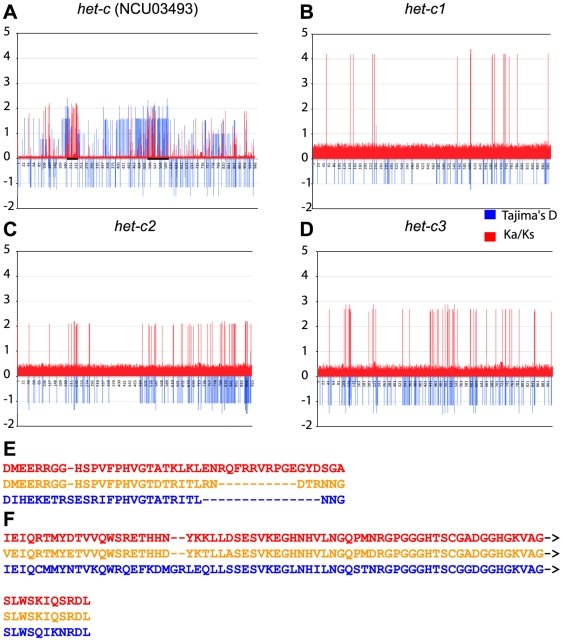
Measure of Ka/Ks ratios and Tajima's D on the coding region of *het-c* show evidence for balancing selection between, but not within allele classes. A) Ka/Ks ratios (ω, ratio of nonsynonymous (Ka) to synonymous (Ks) nucleotide substitution rates; red) calculated for each codon for all *het-c* (NCU03493) alleles shows significant positive selection at positions 126, 215, 219, 232–235, 451 and 546 (P = 0.001) by likelihood ratio test (red).Tajima's D (blue) calculated for each codon shows borderline significant balancing selection (P<0.1) at positions 194–236 (which includes the *het-c* (NCU03493) allelic specificity region [27], [38]). Significant balancing selection (P<0.05) is also seen at positions 521–599. Both regions are shown as bold bars on (A). Evaluation of Ka/Ks (red) and Tajima's D (blue) within a *het-c* (NCU03493) allele class shows evidence for positive selection, but not balancing selection: B) *het-c1* alleles, C) *het-c2* alleles and D) *het-c3* alleles. Note evidence for positive selection at different sites for all three *het-c* (NCU03493) alleles. E and F) Amino acid alignments of known (E) and new putative (F) specificity regions of *het-c* based on Tajima's D analysis. Bold bars on the x-axis of panel A indicate the locations of these regions.

To test for regions of selection in *het-c* (NCU03493) and *pin-c* (NCU03494), Tajima's D was re-calculated on a sliding window of size 3 alignment with a step of 3 columns (corresponding to each codon). The ratio of the rate of non-synonymous substitutions (Ka) to the rate of synonymous substitutions (Ks) or ω was calculated for each codon in a multiple alignment using an evolutionary codon model [51], which enabled calculating ω at each codon site using a maximum-likelihood (ML) approach [52]. For *het-c* alleles, it was clear that balancing selection was confined to two regions of the coding sequence (Figure 6A, E, F). The first of these regions is the well-known specificity domain (codons/amino acids 194–236) [39] (Figure 6E). However, a second region of *het-c* showing balancing selection was also identified (codons/amino acids 521–599) (Figure 6F). These data are consistent with deletion analysis of *het-c*, where constructs missing the specificity domain still retained a low level of incompatibility [38]. Consistent with a negative Tajima's D statistic, Figure 6B–D also show that specific codons of *het-c* appear to be under strong positive selection. These data suggest that selection may be occurring for *het-c* alleles that differ in function, either for restricting induction of PCD between *het-c pin-c* alleles of the same haplotype, or increasing severity of PCD *via* interactions between haplotypes.

**Figure 7:**
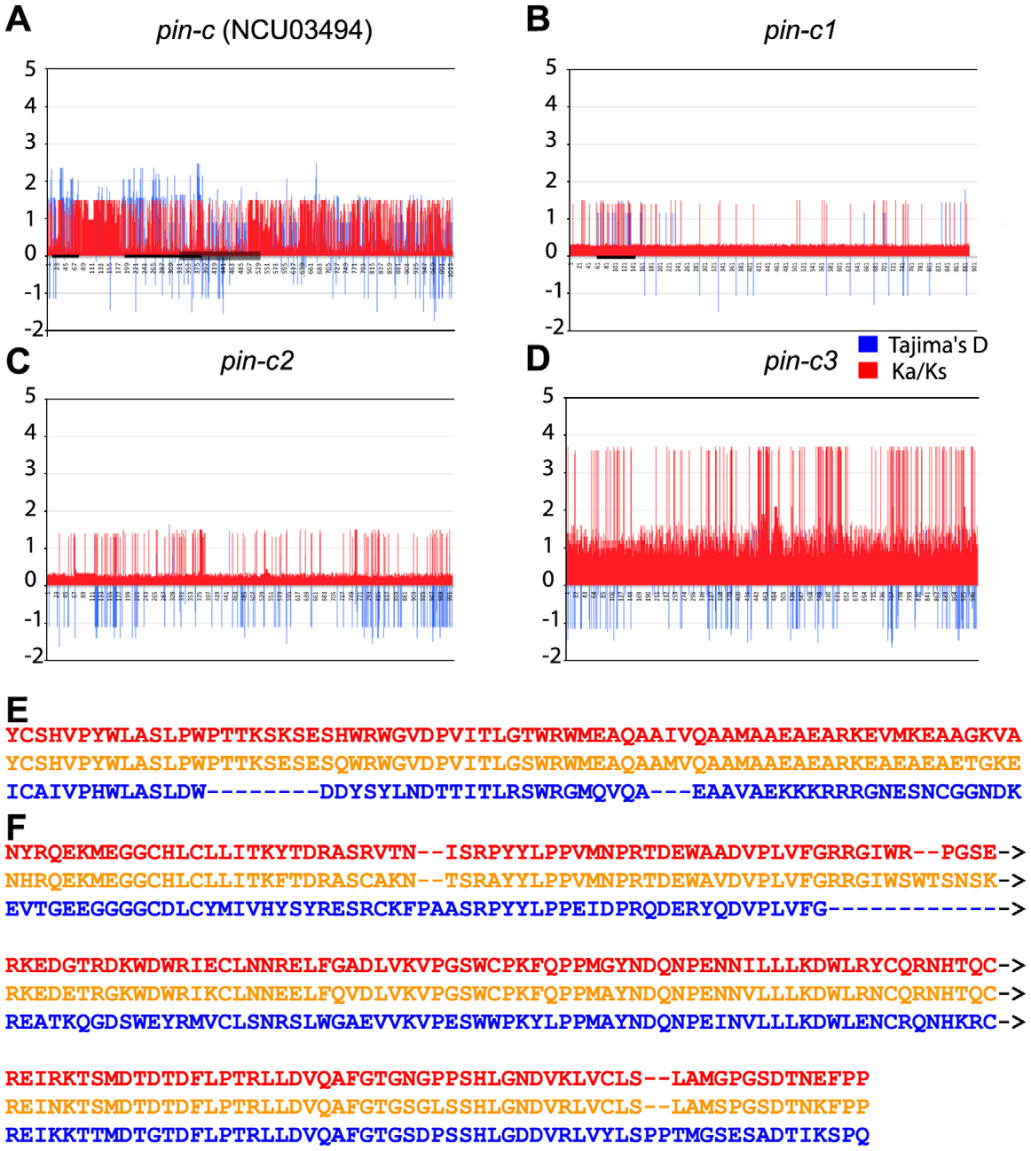
Measure of Ka/Ks ratios and Tajima's D on the coding region of *pin-c* (NCU03494) show evidence for balancing selection between allele classes. Ka/Ks ratios (ω, ratio of nonsynonymous (Ka) to synonymous (Ks) nucleotide substitution rates; red) were calculated for each codon for all *pin-c* alleles and significant positive selection between positions 77–746 (p = 0.001) by likelihood ratio test was detected. These positions include the HET domain (codons 335–533) of *pin-c* (NCU03494)(large bar on x-axis). Tajima's D calculated for each codon for all *pin-c* alleles shows significant balancing selection (P<0.1) at positions 8–78 and 190–390 (smaller bars on x-axis; see E and F). Evaluation of Ka/Ks (red) and Tajima's D (blue) within a *pin-c* allele class shows variable selection: B) A region from codon 60 to 150 shows evidence of balancing selection in *pin-c1* alleles (shown as a bar on the x axis) C) *pin-c2* D) *pin-c3*. *pin-c3* alleles show an extremely high **ω** with three very polymorphic regions (codons 448–491, 569–639 and 738–781). E and F) Amino acid alignments of putative allelic specificity regions of *pin-c* (the locations of these regions are indicated by bars on the x-axis of panel A).

For *pin-c*, two regions (codons/amino acids 8–78 and 190–390) appear to be under balancing selection and are likely to be specificity determinants for non-allelic interactions with *het-c* (Figure 7A, E, F). The second and larger of these two regions overlaps with the HET domain (codons/amino acids 335–533). Similar to full gene analyses above, codon-specific analysis of Tajima's D and ω indicated that the three *pin-c* allele classes are under different evolutionary pressures or have significantly different evolutionary histories. A region in the 5′ end of the coding region of *pin-c1* alleles appears to be under balancing selection, with three different allele types (Figure 7B) and which was different from the two regions identified from the comparison of all *pin-c* alleles. These allele variants were not observed in *pin-c2* or *pin-c3* alleles. Both *pin-c2* and *pin-c3* have a large number of codons with large ω. In *pin-c2*, these codons are mirrored by a negative Tajima's D indicating a high rate of low frequency polymorphism (Table 3; Figure 7C). It seems likely that either many codons in *pin-c2* are under positive or diversifying selection or *pin-c2* alleles have had a recent population expansion. Analysis of *pin-c3* alleles showed an extremely high ω with three regions that were highly polymorphic (Figure 7D). However, these codons have a D statistic close to zero indicating a balance between mutation and genetic drift (Table 3). These data are consistent with *pin-c3* alleles being at equilibrium, with many codons under highly relaxed selection.

As was noted for the recombination block characteristic of *het-c*/*pin-c* haplotypes, the unusual selective pressure evident from Ka/Ks ratios and Tajima's D test does not extend to loci surrounding *het-c* and *pin-c*. For example, a similar analysis of Ka/Ks and Tajima's D performed on *gsl-5* (NCU03492) showed no evidence of selection (Figure S4).

### Both *het-c* and *pin-c* show phylogenetic discordance

In *N. crassa* there should be strong selection against rare recombinants between *het-c/pin-c* haplotypes due to production of self-incompatible progeny. However, initial phylogenetic analysis of the different exons of *het-c* and *pin-c* resulted in inconsistent topologies between trees, suggesting that recombination does occur (Figure 4). We evaluated phylogenetic discordance in *N. crassa* using a test for recombination within alignments of *het-c* and *pin-c* using the likelihood-based selection genetic algorithm (GARD) [53]. GARD identified 3 potential recombination break points with four distinct phylogenetic trees that corresponded to different regions of *het-c*. Only one of the four (at position 491 of the alignment) was determined to be significant by the Kishino-Hasegawa test (KH test) (P-value = 0.00060) [54](Figure S5). In *pin-c*, nine potential recombination break points with 10 distinct phylogenetic trees were identified (Figure S6); all 9 breakpoints were significant (P-value = 0.00180).

**Figure 8:**
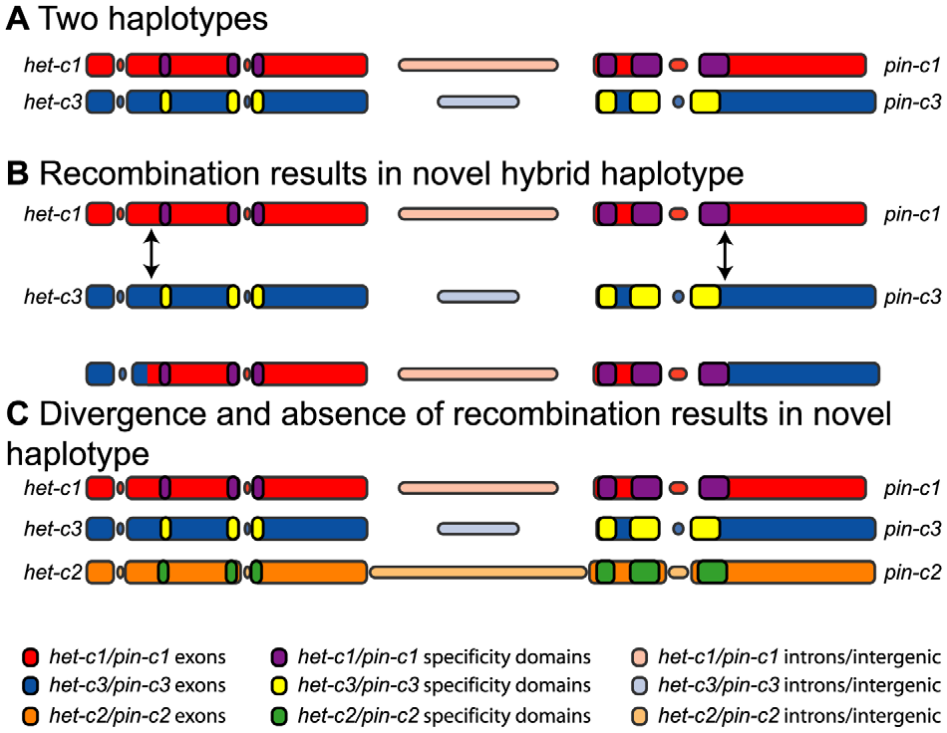
Model of the recombination-based emergence of *het-c2/pin-c2* haplotype. Colored bars indicate the coding and non-coding regions of each haplotype. Red indicates *het-c1/pin-c1*, blue indicates *het-c3/pin-c3*, and yellow indicates *het-c2/pin-c2* haplotypes. Intergenic regions and introns are indicated by thinner bars and muted shades. Indels are show as spaces. The known and putative specificity regions of each haplotype are shown by purple, yellow, and green bars for *het-c1/pin-c1*, *het-c3/pin-c3*, and *het-c2/pin-c2*, respectively. All bars are drawn to scale.

All phylogenetic trees constructed from both *het-c* (NCU03493) and *pin-c* (NCU03494) showed distinct *het-c1/pin-c1* and *het-c3/pin-c3* haplotypes (Figures S5 and S6). However, the *het-c2* and *pin-c2* alleles showed phylogenetic discordance between trees. Most strikingly, *het-c2* alleles were more closely related to *het-c1* alleles, while *pin-c2* alleles were more closely related to *pin-c3* alleles. A close examination of tree topologies revealed that in the first region of *het-c* (nucleotide positions 1–490), *het-c2* alleles form a single unresolved clade that clustered with *het-c3* alleles (Figure S5). In the second region of *het-c* (positions 491–1376), which includes the *het-c* specificity region, the *het-c2* alleles formed a resolved cluster with *het-c1* alleles. This pattern holds through the third region of *het-c* (positions 1377–1899). The fourth region of *het-c* (positions 1900–2832) was poorly resolved. The third and fourth partitions of *het-c* are delineated by poorly supported break points and likely to be evidence of rate variation rather that topological incongruence. However, the sudden topological switch of *het-c2* in regions 1 and 2 indicates that a recombination event occurred between *het-c1* and *het-c3* alleles in the evolution of the *het-c2* allele (Figure 8).

The first and third partitions of *pin-c2* (nucleotide positions 1–222 and 961–1233), cluster with *pin-c1* alleles (Figure S6), consistent with the clustering of *het-c2* with *het-c1* in regions 2–3 (Figure S5). This result was also consistent with the observation that the intergenic regions of *het-c1/pin-c1* and *het-c2/pin-c2*, while highly divergent, showed higher levels of sequence identity (Table 2) and were more closely related to each other than to the intergenic sequence of *het-c3/pin-c3* (Figure S7). The second partition of *pin-c* showed a clearly resolved clade for *pin-c1*, *pin-c2* and *pin-c3*. Interestingly, these partitions overlap closely with the regions determined to be under balancing selection in *pin-c* (Figure 7A). Partition 4 of *pin-c* is largely consistent with partitions 1 and 3 (*pin-c2* clusters with *pin-c1*), but shows some recombination between *pin-c2* and *pin-c3*. However, a sudden topological switch in partition 5 of *pin-c2* (positions 983–1949), and which continues throughout the rest of the gene, results in the clustering of all *pin-c2* alleles with *pin-c3* alleles (Figure S6). This topological shift between regions 3/4 and 5 of *pin-c* is concordant with a similar shift between regions 1 and 2 of *het-c*, consistent with a recombination event occurring between *het-c1/pin-c1* and *het-c3/pin-c3* haplotypes in the evolution of the *het-c2/pin-c2* haplotype (Figure 8).

Our data support a model in which the *het-c2*/*pin-c2* allele has formed recently by recombination between existing *het-c1*/*pin-c1* and *het-c3*/*pin-c3* alleles. By this model, *het-c2*/*pin-c2* diverged from *het-c1*/*pin-c1* and *het-c3*/*pin-c3* quite recently as compared to their divergence time from each other. To explore this further, we estimated divergence times for *het-c* and *pin-c* alleles both from homologous genes in other species and from each other. These estimates were obtained by fitting trees by maximum likelihood (Langely-Fitch) [55] to a molecular clock as implemented in the r8s software [56]. We assumed a constant molecular clock calibrated by a previous estimate of the divergence of the Eurotiomycetes from the Sordariomycetes at 200 million years ago (mya) [34]. Our results indicated a divergence of *Neurospora* from *Sordaria* approximately 21.1 to 31.3 mya and divergence of *N. crassa* and *N. tetrasperma* approximately 2.6 to 2.8 mya. These estimates are very similar to previous estimates of divergence between these groups (36 mya for the divergence of *Neurospora* and *Sordaria* [27] and 3.5 to 5.8 mya for *N. crassa* and *N. tetrasperma* [57]). Consistent with our hypothesis, the *het-c1*/*pin-c1* and *het-c3*/*pin-c3* haplotypes diverged from each other approximately ∼16 to 20 mya, whereas the *het-c2*/*pin-c2* haplotype only appears 3 to 12 mya (Figure S8). This result indicates that the *het-c/pin-c* HI locus was initially bi-allelic. These data also support the gain of *het-c2*/*pin-c2* haplotype in an ancestor of *N. crassa* and *N. tetrasperma* rather that the gain of *het-c2*/*pin-c2* in an ancestor of *N. crassa* and *N. discreta* with subsequent loss of the *het-c2*/*pin-c2* haplotype in *N. discreta*.

Our analysis above indicated that recombination was rare between *het-c/pin-c* haplotypes. Our expectation was that recombination within haplotypes would be unrestricted. To test this hypothesis, we evaluated tree topologies within specific allelic classes for both *het-c* (NCU03493) and *pin-c* (NCU03494) for both coding regions and intergenic sequence. We did not find evidence of recombination within any *het-c* allele class (data not shown), possibly due to the high conservation of *het-c*, which makes any signal of recombination difficult to detect. Similarly, support for recombination within *pin-c1*, or within intergenic regions of a single haplotype, was not detected (data not shown). However, strong evidence for recombination within the *pin-c2* allele class and within the *pin-c3* allele class was detected (Figures S9 and S10); *pin-c2* and *pin-c3* allele classes both contain highly variant strains, which provided robust evidence for recombination.

## Discussion

Here we investigated the evolution and diversity of a nonself recognition locus in *N. crassa*. We show that the *het-c/pin-c* haplotypes that mediate nonself recognition and HI in *N. crassa* have evolutionary features in common with nonself recognition systems in other eukaryotic species, including extreme polymorphism, low recombination frequencies, frequency dependent selection and trans-species polymorphism. Our study shows that the *het-c/pin-c* haplotypes evolved as a consequence of a gene duplication/genomic rearrangement event, whereby *pin-c* was inserted near the *het-c* locus in the ancestor of Sordaria/Neurospora. We show that a third *het-c/pin-c* haplotype (*het-c2/pin-c2*) was generated *via* recombination in an ancestor of *N. crassa*/*N. tetrasperma*. The three *het-c/pin-c* haplotypes subsequently diverged *via* mutation and reduced recombination associated with extreme divergence of the intergenic sequences between *het-c* and *pin-c*. The ability to discriminate nonself *via* genetic differences at *het-c/pin-c* is a gain-of-function consequence of this genome rearrangement/divergence, rather than a disruption of *het-c* or *pin-c* function, as deletion mutants of *het-c* and *pin-c* are phenotypically wild type [26].

*pin-c* alleles are extremely polymorphic (*pin-c* alleles are ∼50% identical and have numerous indels). The *pin-c* gene encodes a cytoplasmic ∼900 amino acid protein with a HET domain (pfam PF06985) [26], [37]. The HET domain is a ∼150 amino acid region that is common in predicted genes in filamentous ascomycete genomes and appears to be uniquely found in these species. The HET domain has no identified function other than for nonself recognition and HI [37]. Six of the seven molecularly characterized *het* interactions involve proteins with predicted HET domains (*N. crassa het-6*, *tol* and *pin-c* [26], [43], [58] and *P. anserina het*-*D*, *het-E* and *het-R* [59], [60]. HET domains are death effectors; over-expression of just the HET domain causes HI and cell death, no matter what the genetic background [61] (our unpublished results). Our data suggest that the gene duplication/genome rearrangement to form *het-c pin-c* haplotypes enabled *pin-c* to function as a death effector upon nonself recognition, perhaps mediated by physical interactions between HET-C and PIN-C proteins. Previously, we showed that physical interaction between alternate HET-C proteins (HET-C1/HET-C2, HET-C1/HET-C3 and HET-C2/HET-C3) was dependent upon the allelic specificity domain [25], [39]. Codon-based analysis of the coding sequence of *het-c* and *pin-c* confirms that the previously identified *het-c* specificity region is under balancing selection (amino acids 194–236). However, we also identified a novel region of *het-c* that was also under balancing selection, and which is consistent with previous experimental results suggesting this region has a role in HI [38]. We also identified two regions under strong balancing selection in *pin-c* (amino acid positions 8–78 and 190–390). These data will enable further experimentation to determine whether these regions are the *pin-c* allelic specificity determinants and whether they mediate protein-protein interactions between HET-C and PIN-C.

Analysis of gene order at *het-c* (NCU03493) has shown that the ancestral state of *het-c* was not as a HI locus. This hypothesis is supported by studies in other species in which *het-c* is not polymorphic [62], [63], [64]. For example, analysis of the *het-c* homolog in *P. anserina*, *hch*, showed that 11 isolates possessed identical alleles. However, the introduction of the *N. crassa het-c2* allele *via* transformation induced an HI-like response [64]. Similarly, the introduction of the *het-c2* allele into *Aspergillus niger* also induced an HI-like response, even though no natural polymorphisms existed at the *A. niger het-c* locus [62].

Our analysis of recombination both within and between alleles at *het-c/pin-c* strongly supports a model in which recombination between the existing *het-c1*/*pin-c1* and *het-c3*/*pin-c3* haplotypes resulted in the creation of the novel *het-c2/pin-c2* haplotype. Tajima's D was negative for *pin-c2* alleles (−1.04141) supporting the hypothesis that *pin-c2* alleles have had a recent population expansion, perhaps following the creation of this novel specificity *via* recombination. Our analyses indicated that the *het-c2/pin-c2* haplotype emerged after the origin of the *het-c1/pin-c1* haplotype in the Sordariaceae. Once the rare viable *het-c2/pin-c2* hybrid haplotype existed, this strain would be incompatible with every other *Neurospora* strain, which presumably conferred a strong selective advantage, until it settled into equilibrium with the other *het-c/pin-c* haplotypes. This is exactly the case today, with each *het-c/pin-c* haplotype found in roughly 30% of the population (Figure S2). The generation of novel specificities by recombination appears to function in other self/nonself recognition systems and is well characterized in the orchestrated site-specific V(D)J system that generates variability in immunoglobulin and T cell receptor proteins in vertebrate systems [65]. In plant species, recombination to produce new specificities at the *S*-locus was first proposed by Fisher [66]. Although recombination has been detected between the kinase domain of SRK and SCR [67], [68] and Kusaba *et al* provided evidence that recombination occurs between alleles of the stigma-specific *S* glycoprotein (*SLG = SCR*) in Brassica [69], novel allelic specificities and co-evolution of the *S* receptor kinase (*SRK*) was not determined. Similarly, in the Solanaceae, sequence support for intragenic recombination at the S-RNase gene was observed in *Petunia inflata* [70]. In *N. crassa*, the generation of novel specificities at *het-c/pin-c* is a very similar situation to the evolution of *S*-locus. Both involve co-evolution of multiple genes or the result may be HI, as in the case of *N. crassa*, or self-fertility, as in the case of the *S*-locus in plants. The recombination event that resulted in the *het-c2/pin-c2* haplotype appears to meet this requirement as it involved regions of both *het-c* and *pin-c* to generate a novel specificity.

By genetic analyses, *N. crassa* is predicted to have at least 11 unlinked *het* loci [23]. However, *N. crassa* has 52 HET domain genes, far more than are predicted to function as nonself recognition loci. Our analyses of *pin-c*-like HET domain genes in the genomes of filamentous fungi show multiple gene duplication events, rapid diversification and gene loss (Figure S1). Muirhead *et al* predicted through simulation that given a strong selection for the maintenance of HI, the maximum number of loci possible will function as *het* loci [71]. This simulation also predicted that unless the number of loci with the potential to function in HI was saturated, then each locus would contain two alleles. For example 3 loci with 2 alleles each provides more diversity than 2 loci with 3 alleles each (2^3^<3^2^). Our understanding of the selective advantage conferred by the maintenance of HI in fungi, as a barrier to the spread of infectious mycoviruses and the prevention of resource plundering by less fit genotypes, would fit a model in which the most fit individual would be one that was incompatible with all other individuals in the population, except with itself. Given such a selective pressure and a large population size, fitness is conferred by maximizing *het* diversity on a genome-wide scale. The recent emergence (in an evolutionary time scale) of the *het-c/pin-c* haplotype subsequent to gene duplication and rearrangement can be interpreted as selection for the maximum number of *het* loci. The rapid expansion of the *het-c2* haplotype to create a tri-allelic *het-c/pin-c* system would seem to indicate that the number of *het* loci in *N. crassa* is near saturation. This hypothesis is supported by other tri-allelic *het* loci in *N. crassa* [72]. It is possible that in filamentous fungi, *het* loci are born out of dynamic genomic regions that allow for gene duplication and re-arrangement beyond the genomic norm. *N. crassa* provides an excellent model by which to investigate the birth, diversification and death of self/nonself recognition loci. The genomic resources available in the filamentous fungi, including genome sequences of a number of fungi related to *N. crassa*, well defined species, population genetics and the recent availability of RNA-Seq data for >100 *N. crassa* individuals from two populations will enable a full genome analysis of evolution and diversification of all nonself recognition loci encoded within a genome.

## Methods

### Strains and culture conditions

In this study, the laboratory strain (FGSC 2489) and 41 additional wild *N. crassa* strains, 9 wild *N. discreta* strains, and 13 wild *N. tetrasperma* strains were used (Table S1). Twenty-six of the *N. crassa* strains and all of the *N. tetrasperma* strains originated from Franklin, Louisiana. The remaining strains originated from collection sites in Hawaii, Louisiana (not Franklin), Montana, New Mexico, Haiti, Ivory Coast, Liberia, Pakistan, and Panama. All of the *N. crassa* and *N. tetrasperma* strains used in this study are part of the Perkins collection [73] administered by the Fungal Genetics Stock Center (FGSC) or from FGSC's own collection. The strains of *N. discreta* used in this study are from the personal collection of David Jacobsen and were acquired from the John Taylor Laboratory at UC Berkeley. All strains from which genomic DNA was sequenced were cultivated on Vogel's minimal media (VMM) [74] on slants and plates at 22° or 34° C by standard methods [74]. Strains from which RNA was sequenced by RNA-Seq were grown on a VMM plate at 25°C for 25 hours under constant light. A plug of hyphae was cut from each plate and transferred to a Bird's media [75] plate overlaid with cellophane. The culture was incubated at 25°C under constant light.

### RNA extraction

Mycelia was harvested and immediately added to 1 mL of TRIzol reagent (Invitrogen Life Technologies) and Zirconia/silica beads (0.2 g, 0.5-mm diameter; Biospec Products). Cells were disrupted using a MiniBeadBeater instrument (Biospec Products) at maximum speed for 30 seconds twice in succession. Total RNA was extracted according to the manufacturer's protocol for TRIzol (Invitrogen). Total RNA was quantified by bioanalyzer (Agilent).

### cDNA synthesis

For polyA RNA purification, 10 µg of total RNA was bound to dynal oligo(dT) magnetic beads (Invitrogen 610.02) two times, using the manufacturer's instructions. Purified polyA RNA was fragmented by metal-ion catalysis [76] using fragmentation reagents from Ambion (AM12450). For first strand cDNA synthesis 1 µg fragmented polyA RNA was incubated with 3 µg random hexamers (Invitrogen 48190-011), and incubated at 65°C for 5 minutes and then transferred to ice. 1st strand buffer (Invitrogen 18064-014) was added to 1× final concentration (4 µL). Dithiothreitol (DTT), dNTPs and RNAseOUT (Invitrogen 10777-019) were added to 100 mM, 10 mM, and 20 U/20 µL respectively, the sample incubated at 25°C for 2 minutes. 200 U of Superserscript II (Invitrogen 18064-014) was added and the sample incubated at 25°C for 10 minutes, 42°C for 50 minutes and 70°C for 15 minutes.

For second strand synthesis, 51 µL of H_2_O, 20 µL of 5× second strand buffer (Invitrogen 10812-014), and dNTPs (10 mM) were added to the first strand cDNA synthesis mix and incubated on ice for 5 minutes. RNaseH (2 U) (Invitrogen 18021-014), DNA pol I (50 U) (Invitrogen 18010-017) were then added and the mixture was incubated at 16°C for 2.5 hours.

### Library construction

End-repair was performed by adding 45µL of H_2_O, T4 DNA ligase buffer with 10 mM ATP (NEB B0202S) (10 µL), dNTP mix (10 mM), T4 DNA polymerase (15 U) (NEB M0203L), Klenow DNA polymerase (5 U) (NEB M0210S), and T4 PNK (50 U) (NEB M0201L) to the sample and incubating at 20°C for 30 minutes. A single base was added each cDNA fragment by adding Klenow buffer (NEB M0212L), dATP (1 mM), and Klenow 3′ to 5′ exo- (15 U) (NEB M0212L). The mixture was then incubated at 37°C in for 30 minutes.

Standard Illumina adapters (FC-102-1003) were ligated to the cDNA fragments using 2× DNA ligase buffer (Enzymatics L603-HC-L), 1 µL of adapters, and DNA ligase (5 U) (Enzymatics L603-HC-L). The sample was incubated at 25°C for 15 minutes.

The sample was purified in a 2% low-melting point agarose gel, and a slice of gel containing 200-bp fragments was removed and the DNA purified. The polymerase chain reaction (PCR) was used to enrich the sequencing library. A 10 µL aliquot of purified cDNA library was amplified by PCR. PCR cycling conditions were: a denaturing step at 98°C for 30 seconds, 14 cycles of 98°C for 10 seconds, 65°C for 30 seconds, 68°C for 30 seconds, and a final extension at 68°C for 5 minutes.

All libraries were sequence using an Illumina Genome Analyzer-II (Vincent J. Coates Genomic Sequencing Laboratory, UC Berkeley) using standard Illumina operating procedures.

### Sequencing

Genomic DNA was extracted from all strains by the standard method of grinding in liquid nitrogen followed by phenol/chloroform purification [77]. Regions spanning the *het-c*/*pin-c* region were amplified by polymerase chain reaction (PCR) using standard methods. Primer sequences are available upon request. The resulting PCR fragments were purified and directly sequenced using ABI dye terminator chemistry using standard methods. Sequences for NCU03492 were amplified by PCR and sequenced by standard methods and by RNA-seq. All sequences have been submitted to Genbank under accession numbers HQ396330-HQ396439.

### Phylogenetic analyses

Sequences used to construct phylogeny of *pin-c* and related genes for Figure 3 and Figure S1 were acquired from GenBank. Gene names are listed in Figure S1. Strain and gene information used to construct Figure 2 are summarized in Table S2. Sequences used for the construction of Figures 4 and 5 were generated in this study. Information on strain/gene name and accession numbers are listed in Table S3.

Alignments were performed using T-coffee [78]. Regions of the alignment containing gaps were removed from the analysis. Models of molecular evolution were selected using the Akaike Information Criterion (AIC) implemented in ProtTest 2.4 [79]. Bayesian AA analysis employed the Whelan-Goldman AA substitution protein matrix [80]. Using MrBayes 3.1.1 [91], two runs were carried out for 1 M generations. Each run included four chains, and trees were sampled every 100 generations. A burnin sample of 1000 trees was discarded. The remaining trees were used to estimate posterior probabilities and branch lengths with the sumt command in MrBayes [91].

### Analysis of Recombination

The Genetic Algorithm Recombination Detection (GARD) method of Kosakovsky Pond *et al* was used to determine recombination break point [53]. Substitution models used in the GARD analysis were chosen for each analysis by comparison to the general time reversible model (GTR) [81] using the information criterion of Akaike [82]. Models used were Tamura-Nei (TrN) [83] for *het-c* (NCU03493) and Hasegawa, Kishino and Yano 1985 (HKY85) [84] for *pin-c* (NCU03494). For *het-c1/pin-c1* intra-allele analyses, sequences used were from strains FGSC 1945 (AF196305.1, DQ309558.1), FGSC 1824 (HQ396342), FGSC 967 (HQ396387), P4452 (HQ396347), P4454 (HQ396333), P4456 (HQ396338), P4464 (HQ396350), P4480 (HQ396432), P4483 (HQ396377), P4487 (HQ396362), P4489 (HQ396422), FGSC 2489 (XM_950787.2, XM_950788.1), P4471 (HQ396391), P4479 (HQ396433), P4484 (HQ396388), P4490 (HQ396421), P4491 (HQ396383), FGSC 2190 (AF195874.1, DQ309557.1), FGSC 1130 (HQ396336), FGSC 4832 (HQ396394), P4448 (HQ396397), P4451 (HQ396357), P4455 (HQ396413), P4476 (HQ396340), P4486 (HQ396363), and P4496 (HQ396379) for *het-c* (NCU03493) and *pin-c* (NCU03494). FGSC 2489 (XM_950787.2, MX_950788.1), P4471 (HQ396391), P4479 (HQ396433), P4484 (HQ396388), P4490 (HQ396421) and P4491 (HQ396383) were used. For *het-c2/pin-c2* intra-allele analyses, sequences from FGSC 2190 (AF195874.1, DQ309557.1), FGSC 1130 (HQ396336), FGSC 4832 (HQ396394), P4448 (HQ396397), P4451 (HQ396357), P4455 (HQ396413), P4476 (HQ396340), P4486 (HQ396363) and P4496 (HQ396379) were used. For the *het-c3/pin-c3* intra-allele analyses, sequences from FGSC 1945 (AF196305.1, DQ309558.1), FGSC 1824 (HQ396342), FGSC 967 (HQ396387), P4452 (HQ396347), P4454 (HQ396333), P4456 (HQ396338), P4464 (HQ396350), P4480 (HQ396432), P4483 (HQ396377), P4487 (HQ396362), and P4489 (HQ396422) were used. The version of GARD used was that implemented on the Datamonkey web server [85] with no site-to-site rate variation and 2 rate classes.

### Coalescent Analysis

We used the Langley-Fitch (LF) [55] method to fit phylogenetic trees for *het-c* (NCU03493) and *pin-c* (NCU03494) to geologic time. We used the LF implementation found in the r8s suit of programs [56]. We assumed a consistent molecular clock across the tree with the root dated at 200 million years ago, the estimated divergence time of the crown Ascomycota [34]. Trees for *het-c* and *pin-c* used in the coalescent analysis were calculated by maximum likelihood (ML) in PAUP* [86]. Alignments were performed using T-coffee [78]. Regions of the alignment containing gaps were removed from the analysis. Models of molecular evolution were selected using the Akaike Information Criterion (AIC) implemented in jModeltest [87]. The ML analysis employed the HKY85 model of sequence evolution was used with the gamma distribution.

### Calculation of Ka/Ks and Tajima's D

Tajima's D [50] statistics and Ka/Ks were calculated for both *het-c* (NCU03493) and *pin-c* (NCU03494) both between and within allele classes for 29 strains of *N. crassa*. Aligned coding sequences of *het-c* (NCU03493) and *pin-c* (NCU03494) from FGSC 967 (*het-c3/pin-c3*, HQ396387), FGSC 1130 (*het-c2/pin-c2*, HQ396336), FGSC 1824 (*het-c3/pin-c3*, HQ396342), FGSC 1945 (*het-c3/pin-c3*, AF196305.1, DQ309558.1), FGSC 2190 (*het-c2/pin-c2*, AF195874.1, DQ309557.1), FGSC 2489 (*het-c1/pin-c1*, DQ309556.1, XM_950787.2), FGSC 4832 (*het-c2/pin-c2*, HQ396394), P4448 (*het-c2/pin-c2*, HQ396397), P4451 (*het-c2/pin-c2*, HQ396357), P4452 (*het-c3/pin-c3*, HQ396347), P4454 (*het-c3/pin-c3*, HQ396333), P4455 (*het-c2/pin-c2*, HQ396413), P4456 (*het-c3/pin-c3*, HQ396338), P4464 (*het-c3/pin-c3*, HQ396350), P4471 (*het-c1/pin-c1*, HQ396391), P4476 (*het-c2/pin-c2*, HQ396340), P4479 (*het-c1/pin-c1*, HQ396433), P4480 (*het-c3/pin-c3*, HQ396432), P4481 (*het-c1/pin-c1*, HQ396401), P4483 (*het-c3/pin-c3*, HQ396377), P4484 (*het-c1/pin-c1*, HQ396388), P4486 (*het-c2/pin-c2*, HQ396363), P4487 (*het-c3/pin-c3*, HQ396362). P4489 (*het-c3/pin-c3*, HQ396422), P4490 (*het-c1/pin-c1*, HQ396421), P4496 (*het-c2/pin-c2*, HQ396379), and P4501 (*het-c3/pin-c3*, HQ396427) were used for both analyses. The D statistic in all cases was calculated by the method of Tajima as implemented in DnaSP [88]. All segregating sites were considered. Two-tailed confidence limits of D were determined assuming a beta distribution as recommended by Tajima. Full length alignments were used to calculate D both within and between alleles. A 3 bp sliding window was used for codon by codon calculations of D.

The ratio of the rate of non-synonymous substitutions (Ka) to the rate of synonymous substitutions (Ks) was calculated for full length *het-c* (NCU03493) and *pin-c* (NCU03494) sequences from the same strains of *N. crassa* used to calculated the D statistic. The Ka/Ks ratio or ω was calculated for each codon in a multiple alignment using an evolutionary codon model [51], which enabled calculating ω at each codon site using a maximum-likelihood (ML) approach. The method used was that of Stern *et al* as implemented in Selecton 2.2 [52].

## Supporting Information

Figure S1 Expanded phylogeny of *pin-c* and related genes.
(1.20 MB TIF)

Figure S2 Diversity at *het-c/pin-c* amongst Louisiana and global isolates of *Neurospora crassa*.
(1.05 MB TIF)

Figure S3 Representation of *het-c/pin-c* intergenic sequences in Neurospora.
(1.15 MB TIF)

Figure S4 Measures of Ka/Ks ratios and Tajima's D on the coding region of *gsl-5* (NCU03492) do not show evidence for balancing or positive selection.
(1.37 MB TIF)

Figure S5 Analysis of recombination at *het-c* in *N. crassa*.
(1.60 MB TIF)

Figure S6 Analysis of recombination at *pin-c* in *N. crassa*.
(3.40 MB TIF)

Figure S7 Analysis of recombination in the *het-c/pin-c* intergenic region in *N. crassa*.
(1.25 MB TIF)

Figure S8 Estimated divergence dates of *het-c* and *pin-c* alleles from related HET domain genes.
(0.90 MB TIF)

Figure S9 Analysis of recombination within haplotypes at *pin-c2* in *N. crassa*.
(1.30 MB TIF)

Figure S10 Analysis of recombination within haplotypes at *pin-c3* in *N. crassa*.
(1.61 MB TIF)

Table S1 Table of strains used in this study.
(0.14 MB DOC)

Table S2 Species, strains, and genes used in the construction of Figure 2.
(0.05 MB DOC)

Table S3 Sequence accessions used in figures 4 (*gsl-5* and NCU03495) and 5 (*het-c* and *pin-c*).
(0.11 MB DOC)
