# Supplementary figures and images for: Evolution and Diversity of a Fungal Self/Nonself Recognition Locus
Source: PLoS One. 2010 Nov 19;5(11):e14055. doi: 10.1371/journal.pone.0014055 (PMC2988816; doi:10.1371/journal.pone.0014055)

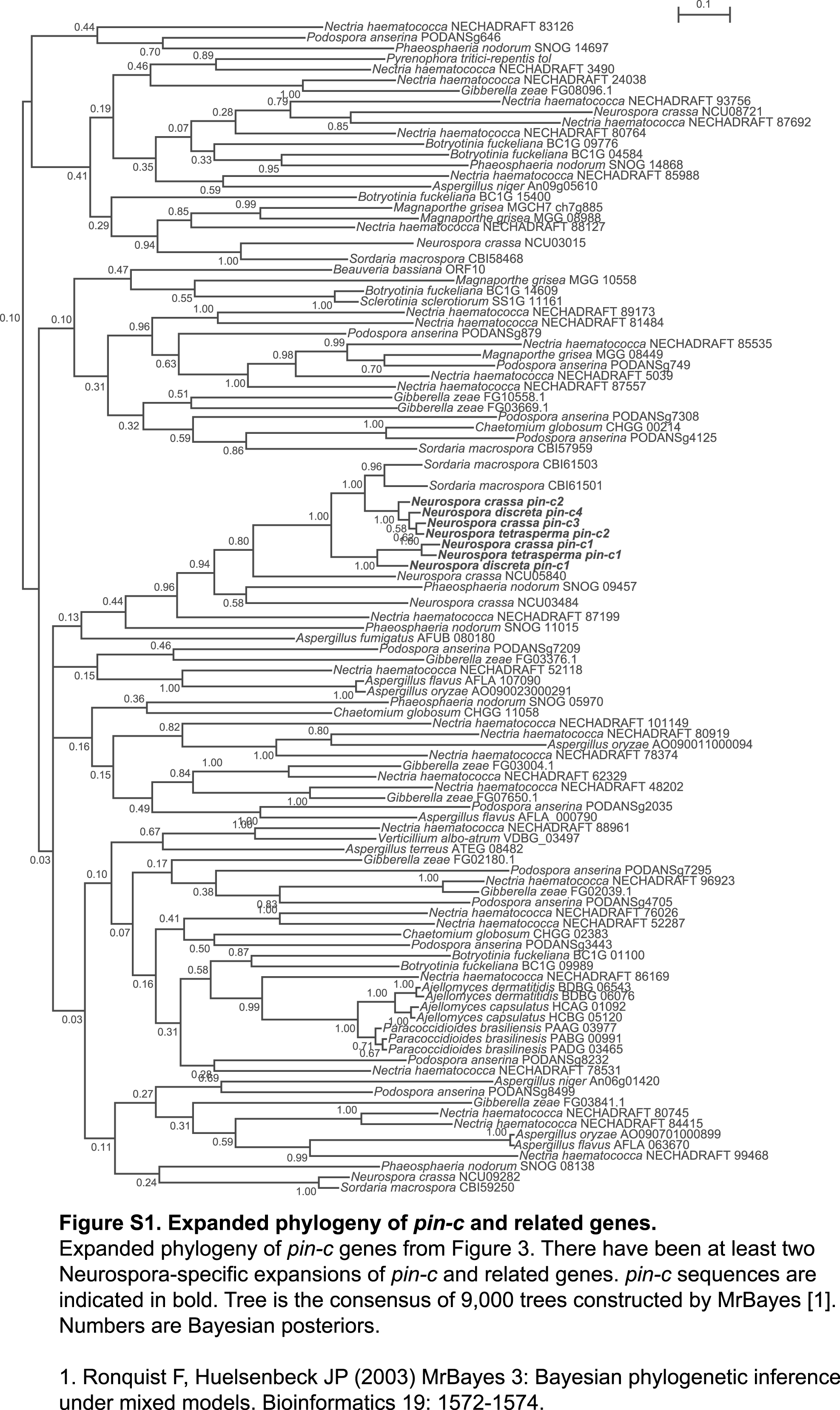

Supplement: Figure S1 — Expanded phylogeny of pin-c and related genes. (1.20 MB TIF) [file pone.0014055.s001.tif]

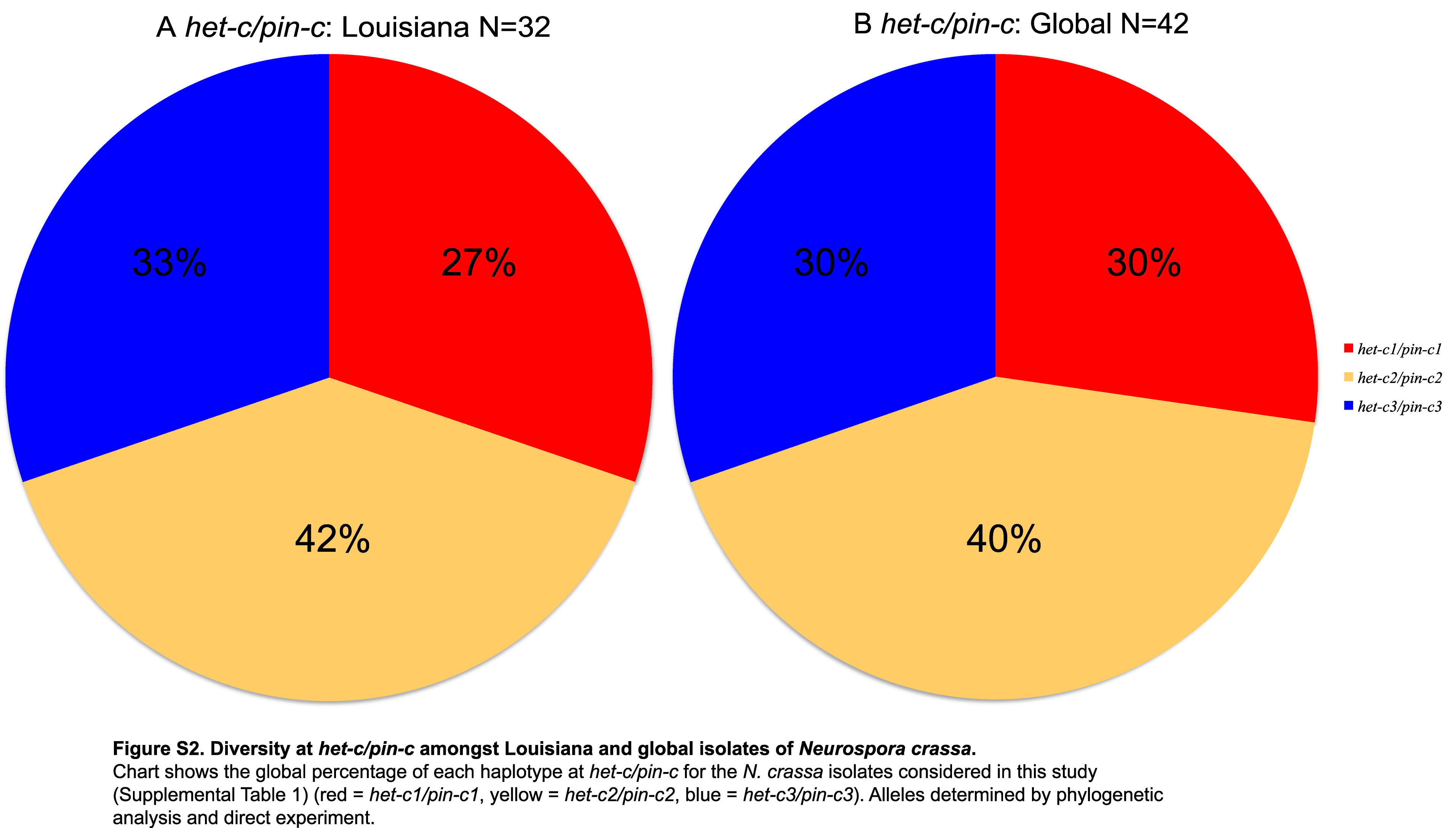

Supplement: Figure S2 — Diversity at het-c/pin-c amongst Louisiana and global isolates of Neurospora crassa. (1.05 MB TIF) [file pone.0014055.s002.tif]

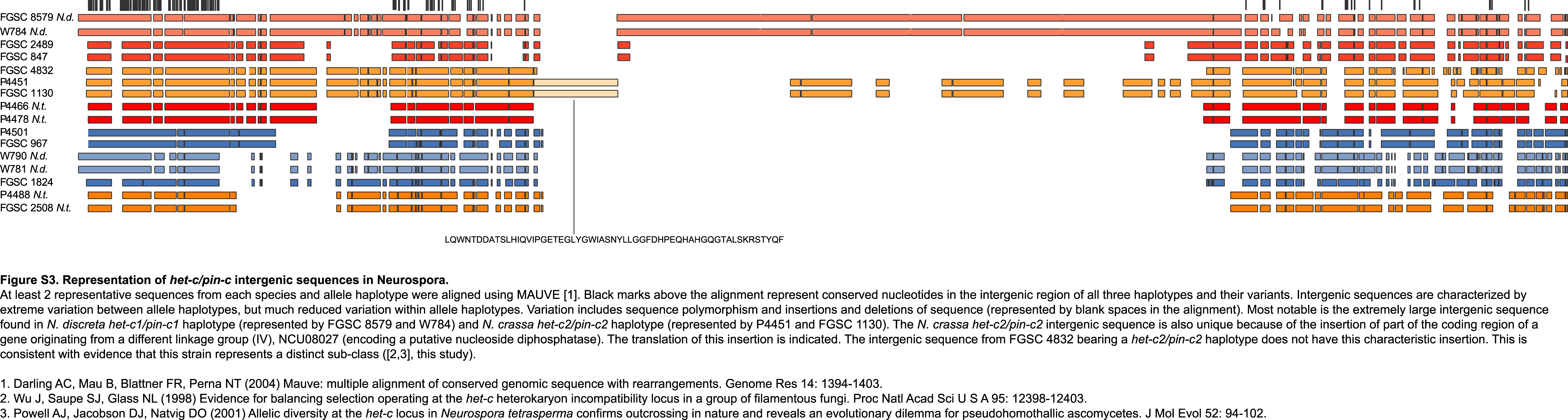

Supplement: Figure S3 — Representation of het-c/pin-c intergenic sequences in Neurospora. (1.15 MB TIF) [file pone.0014055.s003.tif]

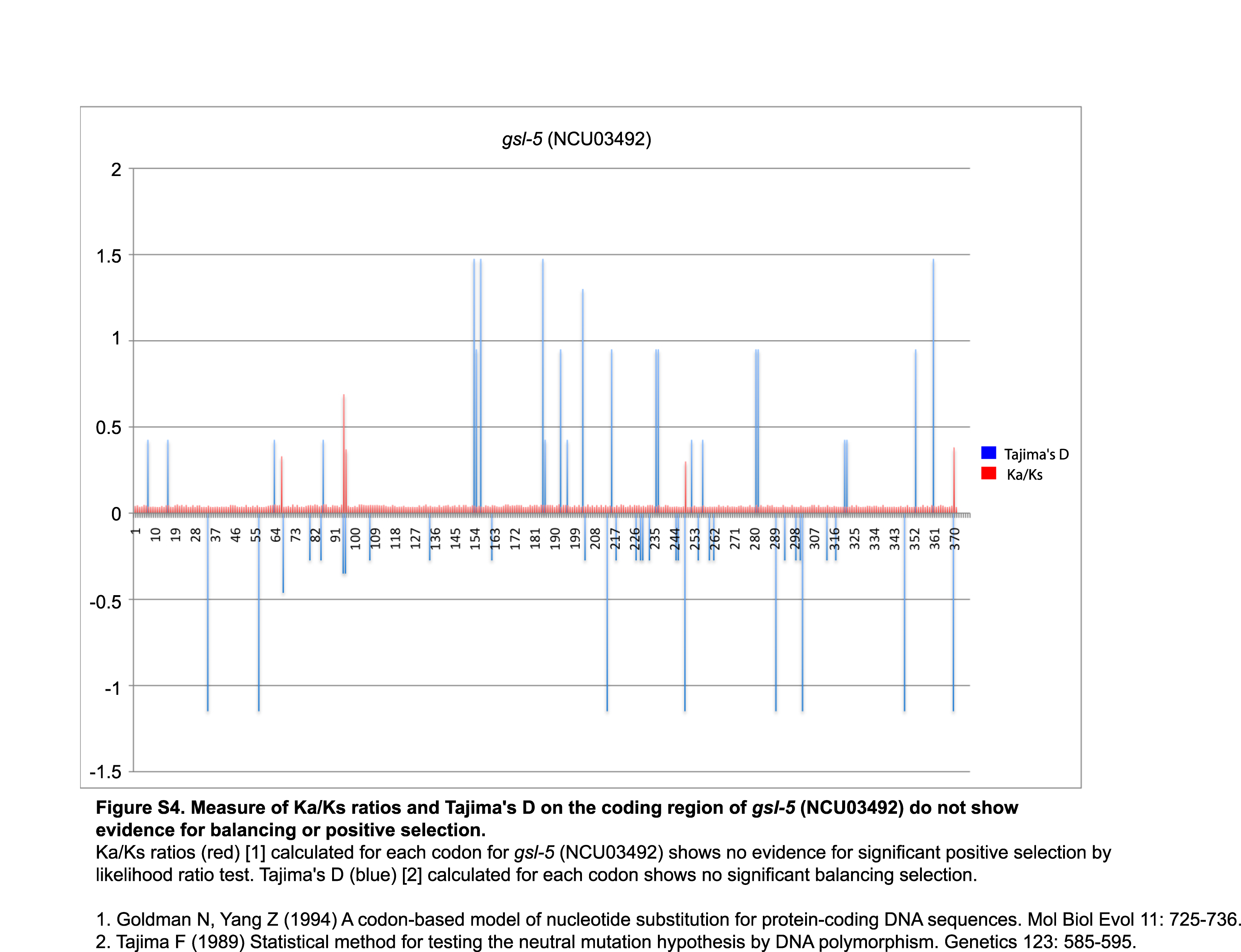

Supplement: Figure S4 — Measures of Ka/Ks ratios and Tajima's D on the coding region of gsl-5 (NCU03492) do not show evidence for balancing or positive selection. (1.37 MB TIF) [file pone.0014055.s004.tif]

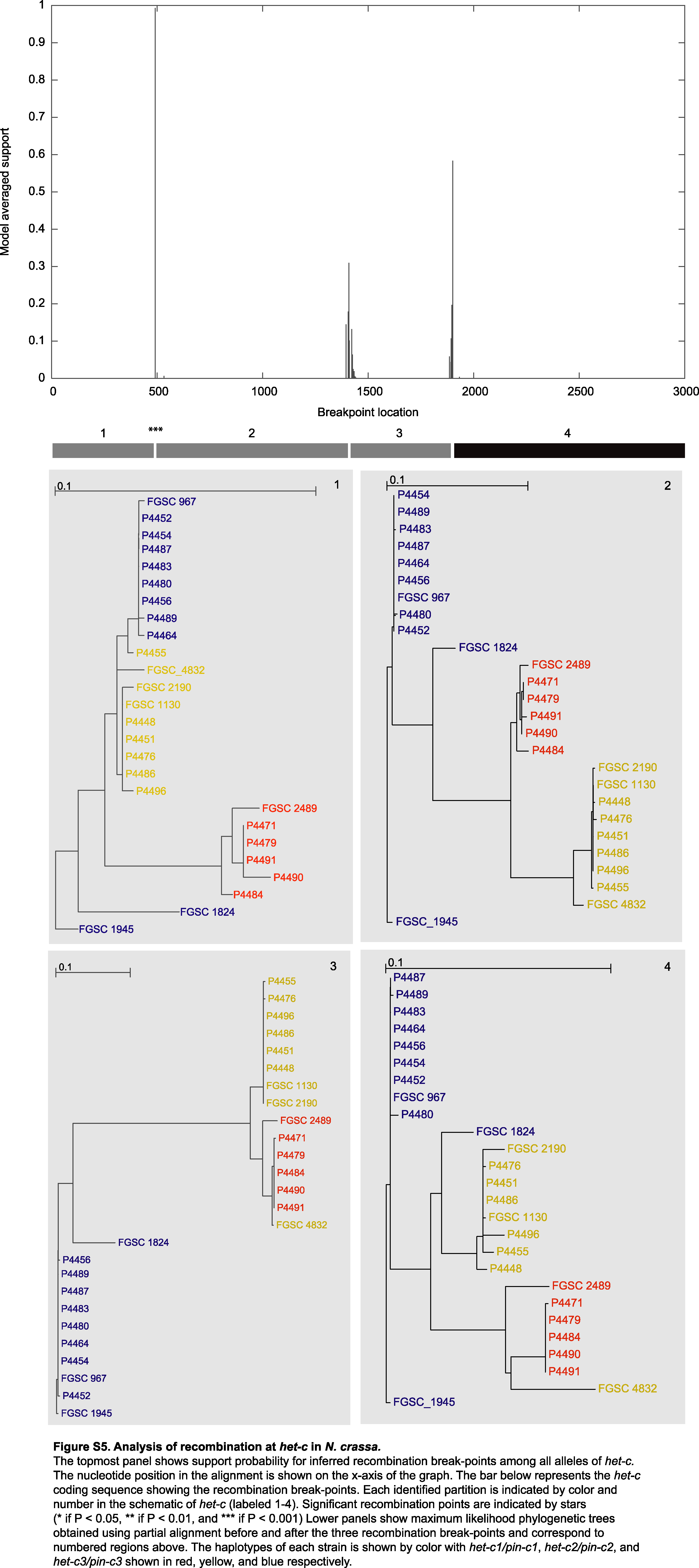

Supplement: Figure S5 — Analysis of recombination at het-c in N. crassa. (1.60 MB TIF) [file pone.0014055.s005.tif]

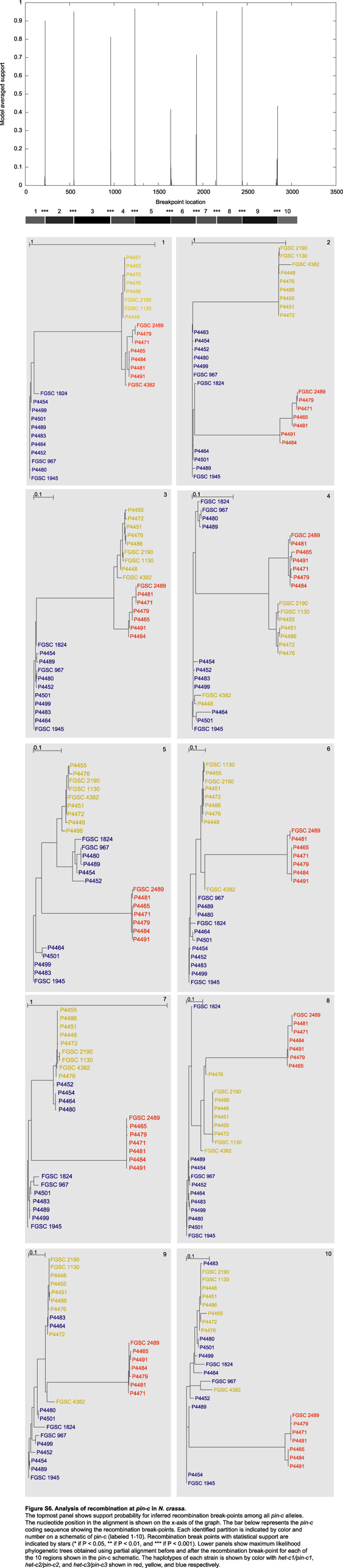

Supplement: Figure S6 — Analysis of recombination at pin-c in N. crassa. (3.40 MB TIF) [file pone.0014055.s006.tif]

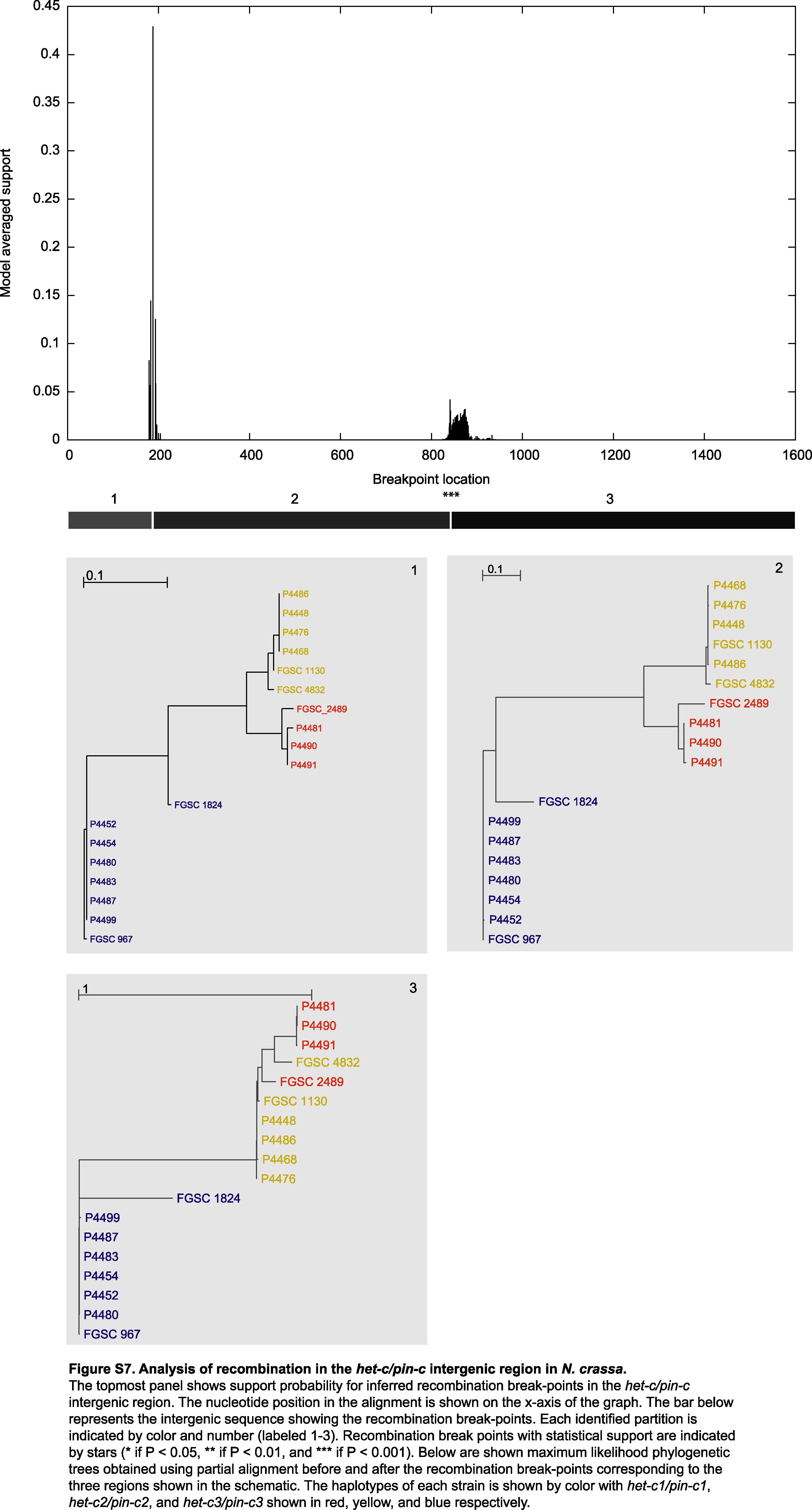

Supplement: Figure S7 — Analysis of recombination in the het-c/pin-c intergenic region in N. crassa. (1.25 MB TIF) [file pone.0014055.s007.tif]

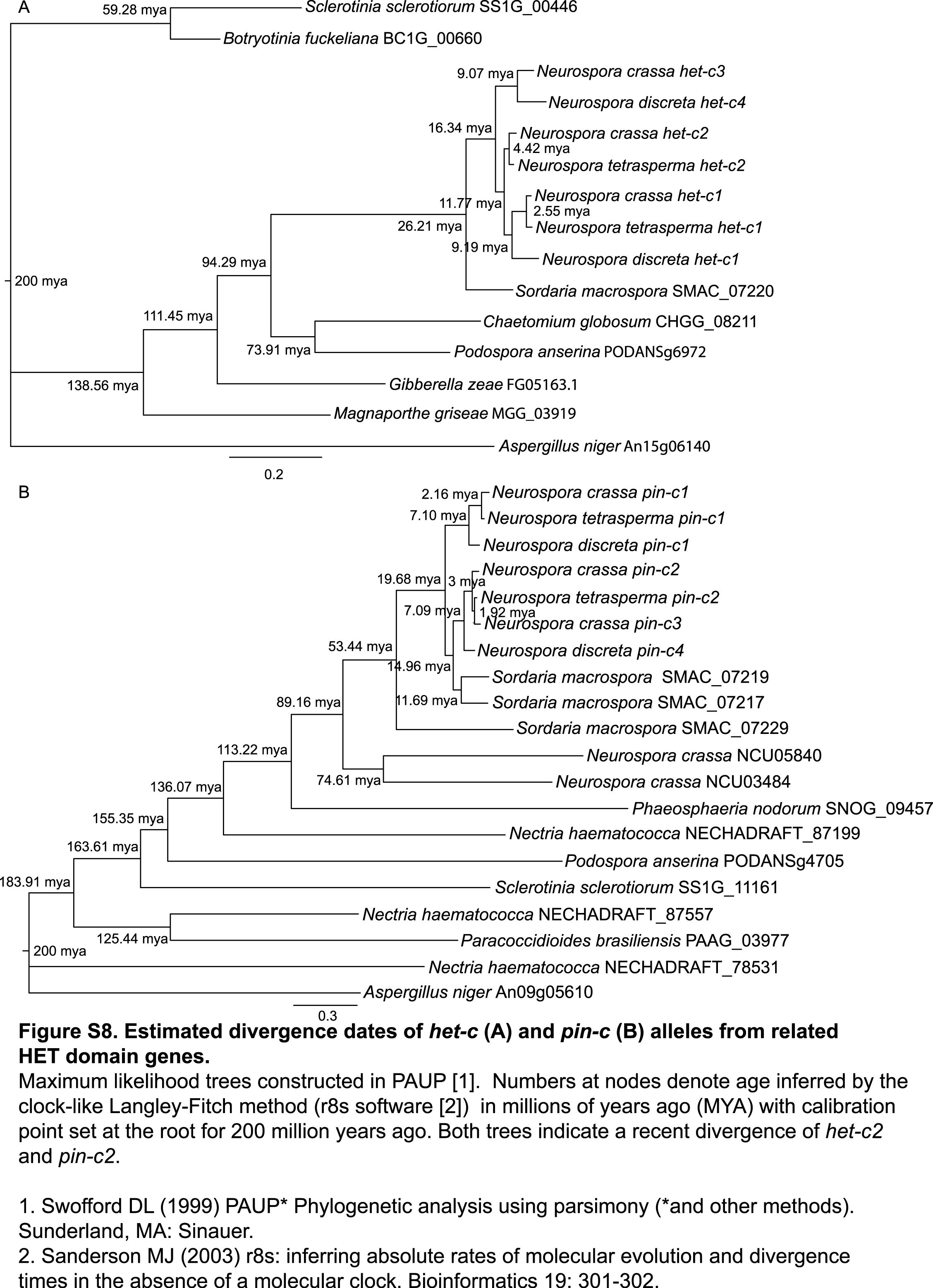

Supplement: Figure S8 — Estimated divergence dates of het-c and pin-c alleles from related HET domain genes. (0.90 MB TIF) [file pone.0014055.s008.tif]

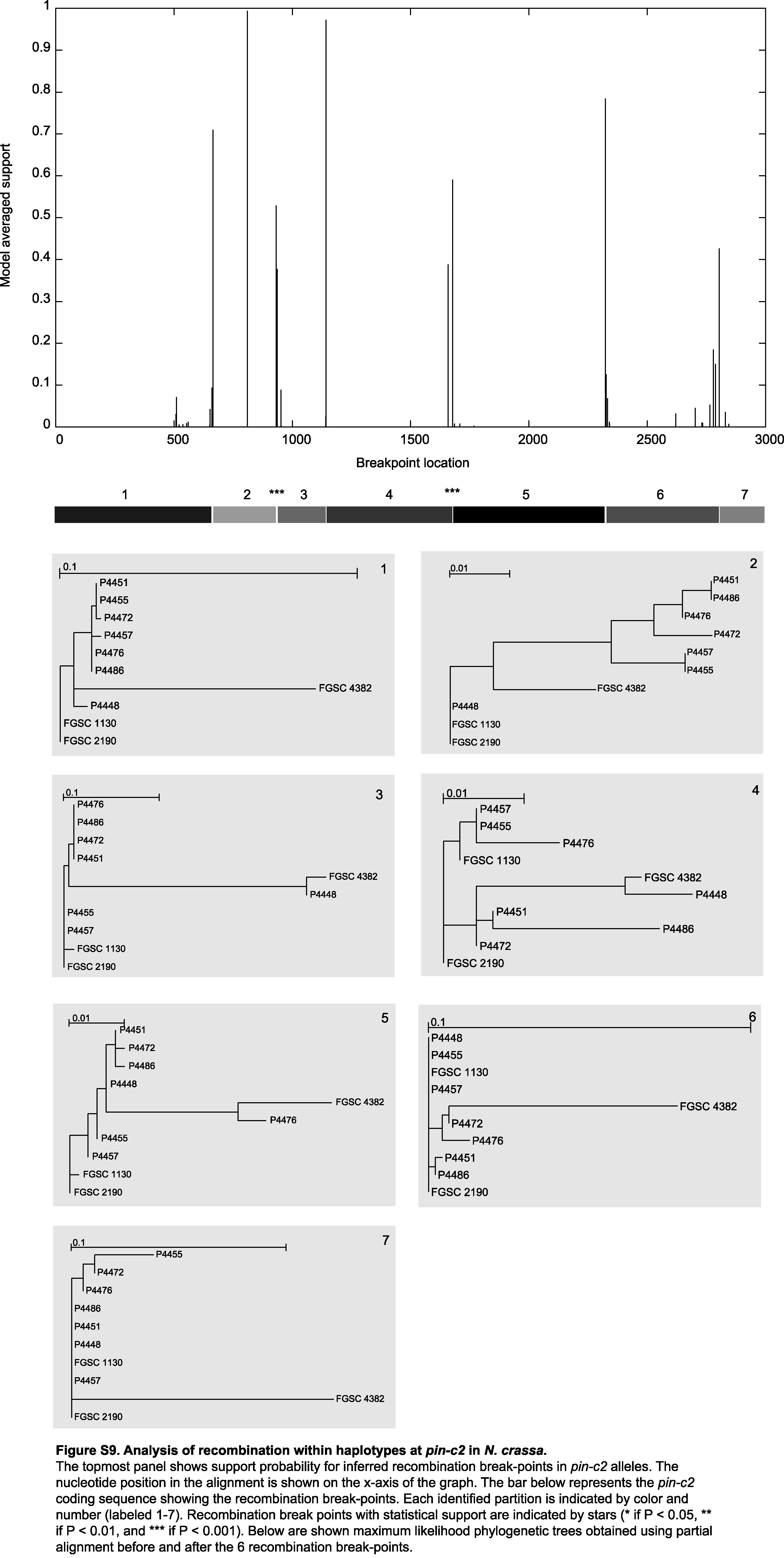

Supplement: Figure S9 — Analysis of recombination within haplotypes at pin-c2 in N. crassa. (1.30 MB TIF) [file pone.0014055.s009.tif]

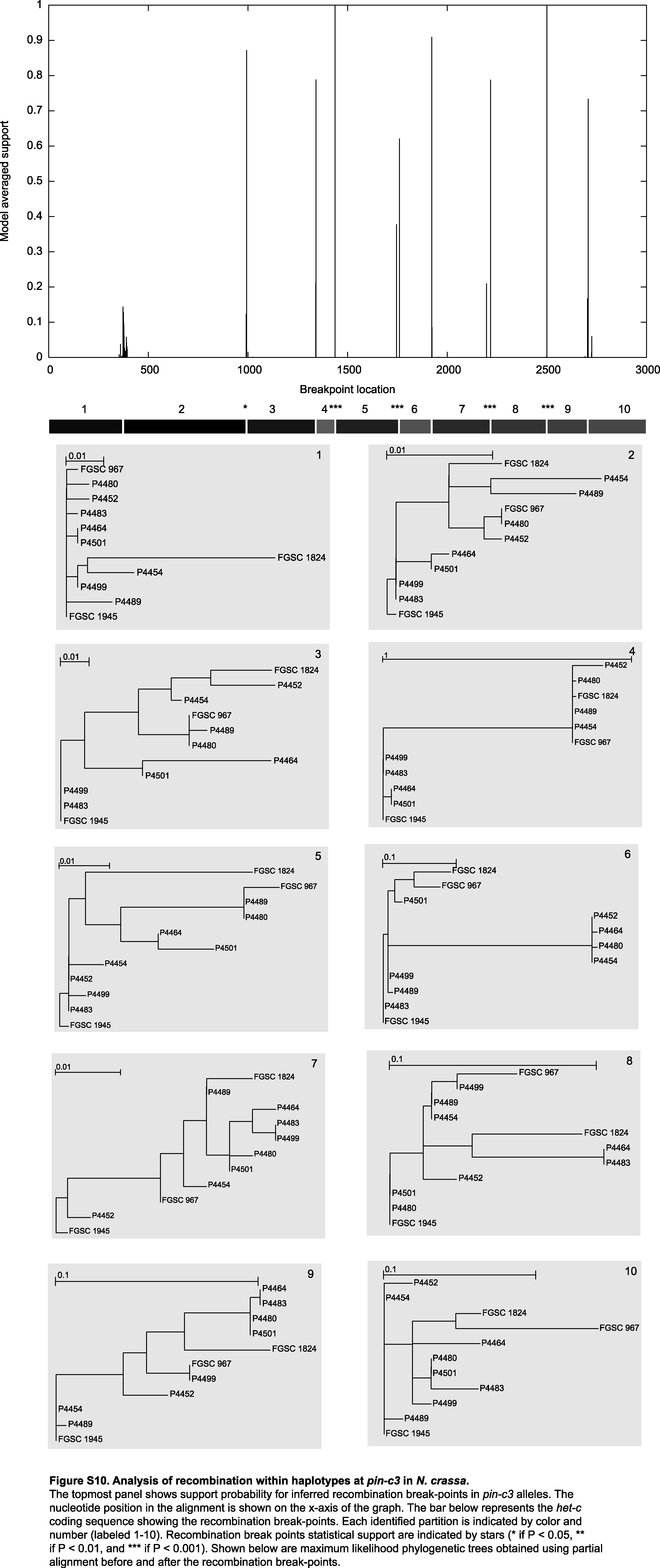

Supplement: Figure S10 — Analysis of recombination within haplotypes at pin-c3 in N. crassa. (1.61 MB TIF) [file pone.0014055.s010.tif]
