# Supplementary material for: Evolution and Diversity of a Fungal Self/Nonself Recognition Locus
Source: PLoS One. 2010 Nov 19;5(11):e14055. doi: 10.1371/journal.pone.0014055 (PMC2988816; doi:10.1371/journal.pone.0014055)
Supplement: Table S1 — Table of strains used in this study. (0.14 MB DOC) [file pone.0014055.s011.doc]

**Supplemental Table 1**. Strains used in this study

| Strain name | Species | Mating type | Origin | Substrate | Depositor | Specificity by tester  cross^4^ |
| --- | --- | --- | --- | --- | --- | --- |
| FGSC 2489^1^ | *N. crassa* | *A* | 74-OR23-1VA | Sequenced strain | OMN | *het-c1* |
| FGSC 847 | *N. crassa* | *A* | Louisiana | Burnt sugarcane | NRRL |  |
| FGSC 967 | *N. crassa* | *a* | Liberia |  | JW | *het-c3* |
| FGSC 1130 | *N. crassa* | *a* | Panama |  | EGS | *het-c2* |
| FGSC 1693 | *N. crassa* | *a* | LA | Burnt sugarcane | ATCC | *het-c1* |
| FGSC 1824 | *N. crassa* | *A* | Pakistan | Burnt grass | DPP | *het-c3* |
| FGSC 1945 | *N. crassa* | *a* | Groveland, FL | Saw palmetto | DPP | *het-c3* |
| FGSC 2190 | *N. crassa* | *a* | FGSC 1131 |  | DPP | *het-c2* |
| FGSC 4709 | *N. crassa* | *a* | Haiti | Burnt grass | DPP | *het-c1* |
| FGSC 4832 | *N. crassa* | *a* | Ivory Coast | Burnt grass | DPP | *het-c2* |
| P4448 | *N. crassa* | *A* | Franklin, LA | Burnt sugarcane | DJJ |  |
| P4449 | *N. crassa* | *a* | Franklin, LA | Burnt sugarcane | DJJ |  |
| P4450 | *N. crassa* | *a* | Franklin, LA | Burnt sugarcane | DJJ | *het-c2* |
| P4451 | *N. crassa* | *a* | Franklin, LA | Burnt sugarcane | DJJ | *het-c2* |
| P4453 | *N. crassa* | *A* | Franklin, LA | Burnt sugarcane | DJJ |  |
| P4454 | *N. crassa* | *a* | Franklin, LA | Burnt sugarcane | DJJ | *het-c3* |
| P4455 | *N. crassa* | *a* | Franklin, LA | Burnt sugarcane | DJJ |  |
| P4456 | *N. crassa* | *a* | Franklin, LA | Burnt sugarcane | DJJ | *het-c3* |
| P4463 | *N. crassa* | *A* | Franklin, LA | Burnt sugarcane | DJJ |  |
| P4464 | *N. crassa* | *A* | Franklin, LA | Burnt sugarcane | DJJ |  |
| P4465 | *N. crassa* | *a* | Franklin, LA | Burnt sugarcane | DJJ |  |
| P4468 | *N. crassa* | *A* | Franklin, LA | Burnt sugarcane | DJJ |  |
| P4469 | *N. crassa* | *a* | Franklin, LA | Burnt sugarcane | DJJ |  |
| P4471 | *N. crassa* | *A* | Franklin, LA | Burnt sugarcane | DJJ |  |
| P4472 | *N. crassa* | *A* | Franklin, LA | Burnt sugarcane | DJJ |  |
| P4476 | *N. crassa* | *A* | Franklin, LA | Burnt sugarcane | DJJ |  |
| P4479 | *N. crassa* | *A* | Franklin, LA | Burnt sugarcane | DJJ |  |
| P4480 | *N. crassa* | *A* | Franklin, LA | Burnt sugarcane | DJJ |  |
| P4481 | *N. crassa* | *A* | Franklin, LA | Burnt  sugarcane | DJJ | *het-c1* |
| P4483 | *N. crassa* | *A* | Franklin, LA | Burnt sugarcane | DJJ | *het-c3* |
| P4484 | *N. crassa* | *A* | Franklin, LA | Burnt  sugarcane | DJJ | *het-c1* |
| P4486 | *N. crassa* | *A* | Franklin, LA | Burnt  sugarcane | DJJ | *het-c2* |
| P4487 | *N. crassa* | *A* | Franklin, LA | Burnt  sugarcane | DJJ |  |
| P4489 | *N. crassa* | *A* | Franklin, LA | Burnt sugarcane | DJJ |  |
| P4490 | *N. crassa* | *A* | Franklin, LA | Burnt sugarcane | DJJ |  |
| P4491 | *N. crassa* | *A* | Franklin, LA | Burnt sugarcane | DJJ | *het-c1* |
| P4494 | *N. crassa* | *A* | Franklin, LA | Burnt sugarcane | DJJ |  |
| P4496 | *N. crassa* | *A* | Franklin, LA | Burnt sugarcane | DJJ |  |
| P4497 | *N. crassa* | *A* | Franklin, LA | Burnt sugarcane | DJJ |  |
| P4498 | *N. crassa* | *A* | Franklin, LA | Burnt sugarcane | DJJ |  |
| P4499 | *N. crassa* | *A* | Franklin, LA | Burnt  sugarcane | DJJ | *het-c3* |
| P4501 | *N. crassa* | *A* | Franklin, LA | Burnt  sugarcane | DJJ |  |
| FGSC 2508^2^ | *N. tetrasperma* | *A* | Lihue-1, HI | grass | DPP |  |
| P4458 | *N. tetrasperma* | *A + a* | Franklin, LA | Burnt sugarcane | DJJ |  |
| P4460 | *N. tetrasperma* | *A + a* | Franklin, LA | Burnt sugarcane | DJJ |  |
| P4461 | *N. tetrasperma* | *A + a* | Franklin, LA | Burnt sugarcane | DJJ |  |
| P4462 | *N. tetrasperma* | *A + a* | Franklin, LA | Burnt sugarcane | DJJ |  |
| P4466 | *N. tetrasperma* | *A + a* | Franklin, LA | Burnt sugarcane | DJJ |  |
| P4473 | *N. tetrasperma* | *A + a* | Franklin, LA | Burnt sugarcane | DJJ |  |
| P4474 | *N. tetrasperma* | *A + a* | Franklin, LA | Burnt sugarcane | DJJ |  |
| P4475 | *N. tetrasperma* | *A + a* | Franklin, LA | Burnt sugarcane | DJJ |  |
| P4477 | *N. tetrasperma* | *A + a* | Franklin, LA | Burnt sugarcane | DJJ |  |
| P4478 | *N. tetrasperma* | *A + a* | Franklin, LA | Burnt sugarcane | DJJ |  |
| P4482 | *N. tetrasperma* | *A + a* | Franklin, LA | Burnt sugarcane | DJJ |  |
| P4488 | *N. tetrasperma* | *A + a* | Franklin, LA | Burnt sugarcane | DJJ |  |
| P4492 | *N. tetrasperma* | *A + a* | Franklin, LA | Burnt sugarcane | DJJ |  |
| FGSC 8579^3^ | *N. discreta* | *A* | Belen, NM | Sequenced Strain | DJJ |  |
| W778 | *N. discreta* | *A* | Bernalillo, NM | Burnt trees | DJJ |  |
| W779 | *N. discreta* | *A* | Bernalillo, NM | Burnt trees | DJJ |  |
| W780 | *N. discreta* | *A* | Bernalillo, NM | Burnt trees | DJJ |  |
| W783 | *N. discreta* | *A* | Bernalillo, NM | Burnt trees | DJJ |  |
| W784 | *N. discreta* | *A* | Bernalillo, NM | Burnt trees | DJJ |  |
| W786 | *N. discreta* | *A* | Bernalillo, NM | Burnt trees | DJJ |  |
| W787 | *N. discreta* | *A* | Bernalillo, NM | Burnt trees | DJJ |  |
| W790 | *N. discreta* | *A* | Bernalillo, NM | Burnt trees | DJJ |  |
| FGSC 1482^4^  FGSC 1483 | *N. crassa* | *A*  *a* | T(II>VR)  NM149 *het-c1* |  | DDP  DDP | *het-c1* tester |
| FGSC 2191  FGSC 2192 | *N. crassa* | *A*  *a* | T(II>VR)  NM149 *het-c2* |  | DDP  DDP | *het-c2* tester |
| FGSC 2193  FGSC 2194 | *N. crassa* | *A*  *a* | T(II>VR)  NM149 *het-c3* |  | DDP  DDP | *het-c3* tester |

^1^ *Neurospora crassa* genome database:

http://www.broadinstitute.org/annotation/genome/neurospora/MultiHome.html

^2^ *Neurospora tetrasperma* database: http://genome.jgi-psf.org/Neute1/Neute1.home.html

^3^ *Neurospora discreta* database: <http://genome.jgi-psf.org/Neudi1/Neudi1.home.html>

^4^ *het-c* specificity of selected strains was determined by crosses with translocation strains according to [1].

1. Perkins DD (1975) The use of duplication-generating rearrangements for studying heterokaryon incompatibility genes in Neurospora. Genetics 80: 87-10**5.**
