# Supplementary material for: Evolution and Diversity of a Fungal Self/Nonself Recognition Locus
Source: PLoS One. 2010 Nov 19;5(11):e14055. doi: 10.1371/journal.pone.0014055 (PMC2988816; doi:10.1371/journal.pone.0014055)
Supplement: Table S2 — Species, strains, and genes used in the construction of Figure 2. (0.05 MB DOC) [file pone.0014055.s012.doc]

Supplemental Table 2. Species, strains, and genes used in the construction of Figure 2.

| **Species and Strain** | **Homologous genes in columns** | | | | | | | | | |
| --- | --- | --- | --- | --- | --- | --- | --- | --- | --- | --- |
| *Neurospora crassa* FGSC 2489 | *pad-1* (NCU03491) | *gsl-5* (NCU03492) | *het-c* (NCU03493) | *pin-c* (NCU03494) |  |  | tRNA (NCU11522) | tRNA (NCU11523) | PP2C (NCU03495) | NCU03496 |
| *Neurospora tetrasperma* FGSC 2508* | *pad-1* (estExt_Genewise1Plus.C_100357) | *gsl-5* (estExt_fgenesh1_pg.C_100151) | *het-c* (estExt_fgenesh1_pm.C_100151) | *pin-c* (estExt_fgenesh1_pg.C_scaffold_10000155) |  |  | tRNA | tRNA | PP2C (estExt_Genewise1.C_100368) | estExt_Genewise1Plus.C_100371 |
| *Neurospora discreta* FGSC 9579* | *pad-1* (estExt_Genewise1Plus.C_160212) | *gsl-5* (estExt_Genewise1.C_160217) | *het-c* (estExt_Genewise1.C_160219) | *pin-c* (estExt_fgenesh2_pg.C_160104) |  |  | tRNA | tRNA | PP2C (estExt_Genewise1.C_160225) | estExt_fgenesh2_pg.C_160106 |
| *Sordaria macrospora* k-hell | *pad-1* (SMAC_07222) | *gsl-5* (SMAC_07221) | *het-c* (SMAC_07220) | *pin-c* (SMAC_07219) | SMAC_07218 | SMAC_07217 | tRNA | tRNA | PP2C (SMAC_07230) | SMAC_07231 |
| *Podospora anserina* DSM 980 | PODANSg6974 | PODANSg6973 | PODANSg6972 | PODANSg879 |  |  |  |  | PODANSg6952 | PODANSg6953 |
| *Chaetomium globosum* CBS 148.51 | CHGG_08209 | CHGG_08210 | CHGG_08211 |  |  |  |  |  | CHGG_08212 | CHGG_08213 |
| *Magnaporthe grisea* 70-15 | MGG_12101 | MGG_03920 | MGG_03919 | MGG_08449 |  |  |  |  | MGG_03918 | MGG_12100 |
| *Gibberella zeae* PH-1 | FG05165 | FG05164 | FG05163 |  |  |  |  |  | FG05162 | FG05161 |
| *Sclerotinia sclerotiorum* 1980 | SS1G00444 | SS1G00445 | SS1G00446 | SS1G111161 |  |  |  |  | SS1G01633 | SS1G00456 |
| *Botryotinia fuckeliana* B05.10 | BC1G00662 | BC1G00661 | BC1G00660 | BC1G14609 |  |  |  |  | BC1G02433 | BC1G00643 |
| *Aspergillus niger* CBS 513.88 | An01g14180 | An01g14200 | An15g06140 |  |  |  |  |  | An01g14160 |  |
| *Neosartorya fischeri* NRRL 181 | *pad-1* (NFIA_009510) | NFIA_009500 | *het-c* (NFIA_091090) |  |  |  |  |  | PP2C (NFIA_009530) | NFIA_009480) |

* Genome sequence for *Neurospora tetrasperma*  <http://genome.jgi-psf.org/Neute1/Neute1.home.html>

* Genome sequence for *Neurospora discreta* <http://genome.jgi-psf.org/Neudi1/Neudi1.home.html>
