# Supplementary material for: Evolution and Diversity of a Fungal Self/Nonself Recognition Locus
Source: PLoS One. 2010 Nov 19;5(11):e14055. doi: 10.1371/journal.pone.0014055 (PMC2988816; doi:10.1371/journal.pone.0014055)
Supplement: Table S3 — Sequence accessions used in figures 4 (gsl-5 and NCU03495) and 5 (het-c and pin-c). (0.11 MB DOC) [file pone.0014055.s013.doc]

Supplemental Table 3. Sequence accessions used in figures 4 (*gsl-5* and NCU03495 and 5 (*het-c* and *pin-c*).

| **Strain** | **Gene or locus** | **Accession Number** |
| --- | --- | --- |
| FGSC 1130[[1]](#endnote-2) | *het-c*/*pin-c* | HQ396336 |
| FGSC 1693[[2]](#endnote-3) | *gsl-5* | HQ396406 |
| FGSC 1693[[3]](#endnote-4) | *het-c* | HQ396410 |
| FGSC 1693[[4]](#endnote-5) | NCU03495 | HQ396355 |
| FGSC 1693[[5]](#endnote-6) | *pin-c* | HQ396361 |
| FGSC 1824 | *het-c*/*pin-c* | HQ396342 |
| FGSC 4709 | *het-c* | HQ396373 |
| FGSC 4709 | *pin-c* | HQ396392 |
| FGSC 4832 | *het-c*/*pin-c* | HQ396394 |
| FGSC 847 | *het-c* | HQ396398 |
| FGSC 847 | *pin-c* | HQ396334 |
| FGSC 967 | *het-c*/*pin-c* | HQ396387 |
| P4448 | *gsl-5* | HQ396420 |
| P4448 | *het-c*/*pin-c* | HQ396397 |
| P4449 | *gsl-5* | HQ396346 |
| P4449 | *het-c* | HQ396349 |
| P4449 | NCU03495 | HQ396332 |
| P4449 | *pin-c* | HQ396353 |
| P4450 | *gsl-5* | HQ396337 |
| P4450 | *het-c*/*pin-c* | HQ396335 |
| P4450 | NCU03495 | HQ396330 |
| P4451 | *gsl-5* | HQ396343 |
| P4451 | *het-c*/*pin-c* | HQ396357 |
| P4452 | *gsl-5* | HQ396348 |
| P4452 | *het-c*/*pin-c* | HQ396347 |
| P4452 | NCU03495 | HQ396428 |
| P4453 | *gsl-5* | HQ396415 |
| P4453 | *het-c* | HQ396393 |
| P4453 | NCU03495 | HQ396402 |
| P4453 | *pin-c* | HQ396351 |
| P4454 | *het-c*/*pin-c* | HQ396333 |
| P4455 | *gsl-5* | HQ396331 |
| P4455 | *het-c*/*pin-c* | HQ396413 |
| P4455 | NCU03495 | HQ396344 |
| P4456 | *het-c*/*pin-c* | HQ396338 |
| P4458 | *het-c*/*pin-c* | HQ396429 |
| P4460 | *het-c* | HQ396367 |
| P4460 | *pin-c* | HQ396380 |
| P4461 | *het-c*/*pin-c* | HQ396390 |
| P4462 | *het-c*/*pin-c* | HQ396372 |
| P4463 | *het-c* | HQ396341 |
| P4463 | *pin-c* | HQ396419 |
| P4464 | *het-c*/*pin-c* | HQ396350 |
| P4465 | *gsl-5* | HQ396430 |
| P4465 | *het-c*/*pin-c* | HQ396358 |
| P4465 | NCU03495 | HQ396418 |
| P4466 | *het-c*/*pin-c* | HQ396431 |
| P4468 | *het-c* | HQ396426 |
| P4468 | *pin-c* | HQ396359 |
| P4469 | *het-c* | HQ396352 |
| P4469 | *pin-c* | HQ396354 |
| P4471 | *het-c*/*pin-c* | HQ396391 |
| P4472 | *gsl-5* | HQ396339 |
| P4472 | *het-c*/*pin-c* | HQ396437 |
| P4472 | NCU03495 | HQ396416 |
| P4473 | *het-c*/*pin-c* | HQ396409 |
| P4474 | *het-c*/*pin-c* | HQ396417 |
| P4475 | *het-c* | HQ396365 |
| P4475 | *pin-c* | HQ396439 |
| P4476 | *gsl-5* | HQ396370 |
| P4476 | *het-c*/*pin-c* | HQ396340 |
| P4476 | NCU03495 | HQ396405 |
| P4477 | *het-c* | HQ396411 |
| P4477 | *het-c*/*pin-c* | HQ396412 |
| P4478 | *het-c* | HQ396356 |
| P4478 | *pin-c* | HQ396438 |
| P4479 | *het-c*/*pin-c* | HQ396433 |
| P4480 | *het-c*/*pin-c* | HQ396432 |
| P4481 | *het-c*/*pin-c* | HQ396401 |
| P4482 | *het-c*/*pin-c* | HQ396400 |
| P4483 | *gsl-5* | HQ396378 |
| P4483 | *het-c*/*pin-c* | HQ396377 |
| P4483 | NCU03495 | HQ396389 |
| P4484 | *het-c*/*pin-c* | HQ396388 |
| P4486 | *het-c*/*pin-c* | HQ396363 |
| P4487 | *het-c*/*pin-c* | HQ396362 |
| P4488 | *het-c* | HQ396360 |
| P4488 | *pin-c* | HQ396414 |
| P4489 | *het-c*/*pin-c* | HQ396422 |
| P4490 | *het-c*/*pin-c* | HQ396421 |
| P4491 | *het-c*/*pin-c* | HQ396383 |
| P4492 | *het-c*/*pin-c* | HQ396382 |
| P4494 | *het-c* | HQ396345 |
| P4494 | *pin-c* | HQ396381 |
| P4496 | *gsl-5* | HQ396366 |
| P4496 | *het-c* | HQ396386 |
| P4496 | NCU03495 | HQ396371 |
| P4496 | *pin-c* | HQ396379 |
| P4497 | *gsl-5* | HQ396407 |
| P4497 | *het-c* | HQ396368 |
| P4497 | NCU03495 | HQ396364 |
| P4497 | *pin-c* | HQ396396 |
| P4497 | *pin-c* | HQ396403 |
| P4498 | *het-c*/*pin-c* | HQ396435 |
| P4499 | *het-c* | HQ396436 |
| P4499 | *pin-c* | HQ396369 |
| P4500 | *gsl-5* | HQ396423 |
| P4500 | *het-c*/*pin-c* | HQ396408 |
| P4500 | NCU03495 | HQ396434 |
| P4501 | *het-c*/*pin-c* | HQ396427 |
| W778 | *het-c* | HQ396376 |
| W778 | *pin-c* | HQ396375 |
| W779 | *het-c*/pinc | HQ396374 |
| W780 | *het-c*/*pin-c* | HQ396399 |
| W783 | *het-c* | HQ396395 |
| W783 | *pin-c* | HQ396385 |
| W784 | *het-c*/*pin-c* | HQ396384 |
| W786 | *het-c*/*pin-c* | HQ396425 |
| W787 | *het-c*/*pin-c* | HQ396424 |
| W790 | *het-c*/*pin-c* | HQ396404 |

1. *het-c/pin-c* indicates that the accession contains the *het-c* coding sequence (with introns) the *pin-c* coding sequence (with introns) and the intergenic sequence. [↑](#endnote-ref-2)
2. *gsl-5* indicates the *gsl-5* (NCU03492) cDNA sequence. [↑](#endnote-ref-3)
3. *het-c* indicates *het-c* (NCU03493) partial coding sequence. [↑](#endnote-ref-4)
4. NCU03495 indicates NCU03495 cDNA sequence. [↑](#endnote-ref-5)
5. *pin-c* indicates *pin-c* partial coding sequence. [↑](#endnote-ref-6)
